# Measures of Adiposity and Risk of Rheumatoid Arthritis in Middle-Aged UK Women: A Prospective Cohort Study

**DOI:** 10.3390/nu17091557

**Published:** 2025-04-30

**Authors:** Yuanyuan Dong, Darren C. Greenwood, Laura J. Hardie, Janet E. Cade

**Affiliations:** 1 Nutritional Epidemiology Group, School of Food Science and Nutrition, University of Leeds, Leeds LS2 9JT, UK; j.e.cade@leeds.ac.uk; 2 Leeds Institute for Data Analytics, Faculty of Medicine and Health, University of Leeds, Leeds LS2 9JT, UK; d.c.greenwood@leeds.ac.uk; 3 Division of Clinical and Population Sciences, Leeds Institute of Cardiovascular and Metabolic Medicine, School of Medicine, University of Leeds, Leeds LS2 9JT, UK; l.j.hardie@leeds.ac.uk

**Keywords:** rheumatoid arthritis, obesity, body mass index, waist circumference, diet quality, waist-to-hip ratio, waist-to-height ratio

## Abstract

**Objectives**: To estimate the association between various indicators of obesity-related health risk and the incidence of rheumatoid arthritis (RA) in a large cohort of women. **Methods**: The UK Women’s Cohort Study is a prospective cohort of 35,372 middle-aged women (aged 35–69 at recruitment) initiated in 1995–1998. Obesity was assessed using body mass index (BMI), waist circumference (WC), waist-to-hip ratio (WHR), waist-to-height ratio (WHtR), categorised according to WHO and NICE guidelines, as well as clothing size. Incident RA cases were identified via Hospital Episode Statistics (HES) linkage up to March 2019. Cox regression models were used to estimate RA risk, adjusting for demographics, reproductive factors, and lifestyle factors. Non-linear associations were examined using restricted cubic splines. **Results**: Among 27,968 eligible subjects with complete data linkage (625,269 person-years of follow-up), there were 255 incident RA cases. Obesity (≥30.0 kg/m^2^) was associated with increased RA risk (HR (95% CI) 1.48 (1.02, 2.17), as were abdominal obesity (WC > 88 cm: 1.58 (1.10, 2.27)), WHR ≥ 0.85 (1.56 (1.03, 2.36)), and WHtR ≥ 0.6 (2.25 (1.34, 3.80)). Each 2.5 kg/m^2^ increase in BMI was associated with a 9% higher risk of RA; each 5 cm increase in WC with 6%; each 0.1 increase in WHR with 20%, and each 0.1 increase in WHtR with 27%. Larger clothing sizes were associated with a greater RA risk: for each onesize increment in blouse size and skirt size, the HRs were 1.13 (95% CI: 1.04, 1.22) and 1.13 (95% CI: 1.05, 1.22), respectively. Notably, skirt size ≥ 20 was associated with a 2.36-fold increased risk of RA. There was evidence of effect modification by weight change and menopausal status in obesity-related RA risk. **Conclusions**: Our findings suggest that managing obesity and central adiposity in middle-aged women may be associated with the risk of developing RA. WHtR may serve as a practical alternative to BMI in assessing RA risk. Clothing size, particularly skirt size, could provide a simple, cost-effective proxy for identifying at high risk of RA.

## 1. Introduction

Rheumatoid arthritis (RA) is the most common inflammatory arthritis, affecting approximately 17.6 million people worldwide [1]. Middle-aged women are disproportionately affected and are more likely to suffer from mechanical pain and fibromyalgia [2]. Obesity, a major modifiable risk factor for various chronic diseases, is highly prevalent in this demographic, with 69% of women aged 55 to 74 in the UK classified as overweight or obese [3]. Given the increasing burden of obesity, understanding its role in RA development is crucial for targeted prevention strategies.

Recent scientific consensus has highlighted the limitations of body mass index (BMI) as a measure of obesity, particularly its inability to account for the distribution of body fat and total fat mass, which is strongly associated with metabolic and inflammatory pathways [4]. Alternative anthropometric indices, such as waist circumference (WC), waist-to-hip ratio (WHR), and waist-to-height ratio (WHtR), provide a more precise assessment of obesity-related health risks, as they better capture visceral fat distribution and body shape [4]. These indices have been shown to improve early identification of individuals at metabolic risk, account for population diversity and sex, and enhance targeted interventions for conditions such as cardiovascular disease (CVD) and type 2 diabetes [5]. Similarly, the National Institute for Health and Care Excellence in the UK (NICE) guidelines have suggested that WHtR could be a replacement for obesity measurement [6].

Although public health and clinical advantages of alternatives to BMI are well known, few studies have investigated their role in RA development. Previous epidemiological studies examining the relationship between obesity and RA risk have relied on BMI-based classification, with inconsistent findings. Two meta-analyses, primarily based on case-control studies, showed that being overweight or obese is associated with a higher risk of RA [7,8]; other studies reported no evidence of any association [9,10]. One possible reason could be that these studies overlooked the role of body fat distribution, which could be distinct across regions and sexes. Limited evidence suggests that WC could be more strongly associated with the risk of RA [11], particularly seropositive RA [12].

However, existing studies have not yet examined whether individuals with obesity or abdominal obesity can lower their RA risk by adopting a healthier lifestyle. To our knowledge, it remains unclear whether significant weight changes modify the association between obesity and RA risk, particularly in individuals with high visceral adiposity. Addressing this gap is critical for informing evidence-based public health strategies. The United Kingdom Women’s Cohort Study (UKWCS) provides a unique opportunity to investigate this question in a large, prospective sample of middle-aged UK women. Our study aims to assess the association between multiple anthropometric indices (e.g., BMI, WC, WHR, WHtR, clothing size) and RA risk, while investigating the potential modifying effects of weight change and lifestyle factors.

## 2. Materials and Methods

We followed the Strengthening the Reporting of Observational Studies in Epidemiology—Nutritional Epidemiology (STROBE-nut) guidelines for the reporting of cohort studies (Supplementary Table S1) [13].

### 2.1. Study Design and Participants

We used data from the UKWCS, a large-scale prospective cohort with 35,372 women aged 35–69 years enrolled at baseline [14]. In brief, 500,000 women from England, Scotland, and Wales, who responded to a direct mail questionnaire from the World Cancer Research Fund (WCRF), were eligible for inclusion in the UKWCS. All participants completed a postal questionnaire that collected dietary, lifestyle, demographic, and anthropometric data at recruitment (1995–1998). Individuals living outside of England (*n* = 2314) and those without a National Health Service number to facilitate data linkage (*n* = 695) were excluded. We also excluded participants who had a diagnosis of RA before or at recruitment (*n* = 38), had outlier anthropometric index (BMI < 10 or >60 kg/m^2^, or waist < 50 or >140 cm, or hip < 60 or >160 cm, or weight < 30 or >200 kg, *n* = 160), and those with missing data for anthropometric index (*n* = 1302). After exclusion, a sample size of 30,863 participants remained eligible for this analysis (Supplementary Figure S1).

The study protocol was approved by the National Research Ethics Service Committee for Yorkshire & the Humber—Leeds East (reference 15/YH/0027) in 1993 and was updated in 2017 to include linkage to Hospital Episode Statistics (HES) data (reference 17/YH/0144).

### 2.2. Anthropometric Assessments

Anthropometric indices were derived from self-reported data collected during the baseline survey, following detailed guidance and standardised procedures provided to all participants. Measurements included body weight (kg), height (m), WC (cm), hip circumference (cm), and clothing size (blouse and skirt). These data were used to calculate key adiposity indicators: BMI as body weight (kg) divided by height squared (m^2^); WHR as WC (cm) divided by hip circumference (cm); and WHtR as WC (cm) divided by height (cm). Further details are provided in Supplementary Table S2.

Clothing size has previously been associated with central adiposity and used to predict chronic disease risk [15]. It is considered a practical alternative when direct anthropometric measurements (e.g., WC or hip circumference) are missing or incomplete, and it enhances the assessment of body shape in self-reported surveys.

### 2.3. Assessment of Lifestyle Factors and Other Covariates

Lifestyle factors, including smoking, drinking, physical activity, dietary quality, and energy intake, were obtained through the baseline questionnaire. Smoking status and alcohol consumption were evaluated through frequency, type, and daily quantity. Physical activity was provided using metabolic equivalent tasks (METs), calculated as MET-hours per week by multiplying the duration of activities by their respective MET values [16]. Dietary quality was measured using a validated 217-item food frequency questionnaire (FFQ), which evaluated the intakes of fruits, vegetables, whole grains, nuts and legumes, fish and long-chain omega-3 fatty acids, red and processed meats, sugar-sweetened beverages and fruit juices, trans fat, alcohol, and sodium. Each component was scored from 0 to 10, and the Alternative Healthy Eating Index (AHEI)-2010 score (ranging from 0 to 110) was derived as the sum of these scores, with higher values indicating a healthier diet [17]. Total energy intake was estimated from portion sizes reported in the FFQ and linked to nutrient composition tables (McCance and Widdowson’s Composition of Foods database), reflecting the caloric content of consumed foods rather than their nutritional quality.

Weight change was computed as the difference between weight at enrolment and weight at early adulthood (age 20), and categorised into weight loss (<−2.5 kg), stable weight (−2.5 to 2.5 kg), and weight gain (moderate: 2.5  to 10 kg; large: >10 kg). Additional covariates included age (continuous, years); socio-economic status (SES; categorical: routine/manual, intermediate, professional/managerial); marital status (categorical: married/living as married, separated/divorced, single/widowed); menopausal status (binary: premenopausal, postmenopausal), hormone replacement therapy (HRT; binary: yes, no); and the prevalence of CVD, cancer, or diabetes at recruitment (binary: yes, no). Details on covariate definitions and assessments are provided in Supplementary Table S3.

### 2.4. Ascertainment of RA Cases and Mortality from CVD and Cancer

The primary outcome was RA incidence (International Classification of Diseases, ICD-10 codes M05–M06), ascertained through linkage to HES data up to 31 March 2019. The secondary outcome was mortality from CVD or cancer, obtained through linkage to the National Health Service Central Register (NHSCR) and the Health and Social Care Information Centre (HSCIC), now NHS Digital. Death data were classified according to ICD-9 and ICD-10 codes. The first recorded event (either RA diagnosis or mortality from CVD/cancer) from the combined data sources was used as the endpoint in the time-to-event analysis.

### 2.5. Statistical Methods

All statistical methods were registered in advance on ClinicalTrials.gov (NCT06670144).

Participant characteristics were summarised using descriptive statistics, with means or percentages calculated by obesity indicator group. Cox proportional hazards (PH) regression models, stratified by age group, were used to estimate hazard ratios (HRs) and 95% confidence intervals (CIs) for BMI, WC, WHR, WHtR, blouse and skirt size in relation to RA incidence. All exposures were modelled as continuous variables to assess the linear relationships with RA risk. We also used WHO and NICE guidelines as the basis for classification: BMI (18.5–<25.0, 25.0–<30.0, ≥30.0 kg/m^2^), WC (≤88, >88 cm), WHR (<0.85, ≥0.85), WHtR (<0.5, 0.5 to 0.6, and ≥0.6). Since the sample size of blouse and skirt sizes allowed for a more detailed investigation, the participants were divided into six garment size groups (≤10, 12, 14, 16, 18, ≥20). Reference groups included normal weight (BMI: 18.5–<25.0 kg/m^2^), abdominal leanness (WC ≤ 88 cm), WHR < 0.85, WHtR < 0.5, and blouse/skirt size ≤ UK 10, which were assumed to have the lowest risk.

The person-years were calculated from enrolment to the earliest of the following events: RA diagnosis, censorship due to CVD- or cancer-related death, or the end of follow-up, using age as the timescale. Adjustment for potential confounders was applied progressively in three steps: Model 1 was unadjusted; Model 2 adjusted for age, SES, marital status, menopausal status, HRT, and prevalence of chronic diseases; Model 3 further adjusted for lifestyle factors, including smoking status, alcohol consumption, dietary quality (AHEI-2010 score) and energy intake, physical activity (METs). The PH assumptions were checked using Schoenfeld residuals (Supplementary Table S4).

To estimate the effect of weight change and age on these associations, we added the weight change group (<2.5, −2.5 to 2.5, 2.5 to 10, >10 kg) and age group (≤55, >55 years) to the fully-adjusted model as an interaction term. Further exploratory analyses included testing for interaction effects of lifestyle factors: dietary quality (AHEI-2010 tertiles: low, moderate, high), physical activity (METs: <600, 600–1500, >1500 min/week), and current smoker (yes, no). In addition, we conducted subgroup analysis by menopausal status (premenopausal, postmenopausal) to investigate its modifying effect among middle-aged women. The adjustment set in each subgroup analysis omitted the potential effect modifier.

We conducted a sensitivity analysis to test the robustness of the main results: (1) excluding participants with a survival time < 5 years to check for reverse causation; (2) excluding current smokers, given its known effect on RA risk; (3) excluding participants who developed chronic diseases (e.g., CVD, cancer, or diabetes); (4) excluding participants with weight change greater than 10 kg to minimise the influence of subclinical conditions; (5) additionally adjusting the model for vitamin D intake; (6) applying a competing risk regression model (Fine-Gray method), considering death as a competing event. All statistical analyses were performed using Stata (version 18).

## 3. Results

Of the 30,863 participants potentially eligible in the UKWCS, we excluded those with missing data for marital status (*n* = 280), smoking status (*n* = 382), menopausal status (*n* = 438), physical activity (*n* = 1389) or SES (*n* = 406), leaving 27,968 middle-aged women for analyses. The study flowchart is provided in Supplementary Figure S1.

### 3.1. Characteristics of Included Participants

Over a median follow-up of 22.3 years (625,269 person-years), 255 incident RA cases (1% of this cohort) were identified. At baseline, participants had a mean age of 51.9 years (standard deviation [SD], 9.2). Mean BMI increased from 21.4 kg/m^2^ (SD 2.9) at age 20 to 24.4 kg/m^2^ (SD 4.2) at recruitment. Compared to non-RA participants, those diagnosed with RA were more likely to be older, have lower educational attainment; hold non-professional occupations; be unmarried; use HRT; and have a higher prevalence of chronic disease. RA cases also exhibited higher baseline BMI and waist circumference, lower physical activity levels, and were more likely to be current or former smokers, with slightly lower alcohol consumption compared to non-cases, though total energy intake and AHEI-2010 scores were comparable between the two groups. Table 1 summarises these characteristics by RA incidence among the 27,968 participants.

Baseline characteristics of the 27,968 participants by indicators of obesity-related health are presented in Supplementary Tables S5 and S6. Different adiposity indicators showed consistent associations with demographic and lifestyle factors. Specifically, participants with higher BMI, WC, WHR, WHtR, and larger blouse or skirt size were more likely to be older, postmenopausal, have lower education levels, be unmarried, and hold non-professional occupations. Also, chronic diseases and HRT use were more prevalent among participants with higher adiposity, with the highest occurrence observed in those with a WHtR ≥ 0.6 (*n* = 115 [15.6%]). Physical activity levels were highest in participants with a skirt size ≤ 10 (17.7 h/week, SD 12.0), a blouse size ≤ 10 (17.1 h/week, SD 11.5), and those in the normal weight group (16.8 h/week, SD 11.4), and lowest among individuals living with obesity (BMI ≥ 30 kg/m^2^, 15.8 h/week, SD 12.9), a WHtR ≥ 0.6 (15.8 h/week, SD 12.6), and a skirt size ≥ 20 (15.4 h/week, SD 12.0). Participants with higher BMI, WC, WHR, WHtR, blouse size, and skirt size were generally less likely to smoke, consume alcohol, or have a high diet quality. Energy intake was highest in the group with a WHR of 0.80–0.85 (2355 kcal/day, SD 697.8) and lowest among those with a skirt size ≤ 10 (2287 kcal/day, SD 714.8).

### 3.2. Associations of BMI, WC, WHR, WHtR, Blouse Size, Skirt Size with RA Risk

**Figure 1:**
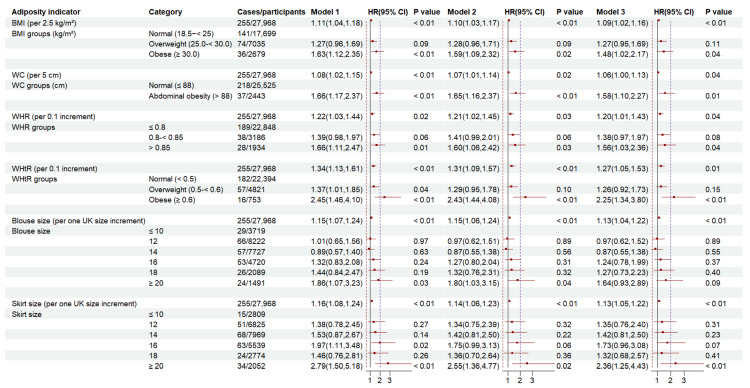
Association between indicators of adiposity and risk of rheumatoid arthritis in the UK Women’s Cohort Study. BMI, body mass index; WC, waist circumference; WHR, waist-to-hip ratio; WHtR, waist-to-height ratio; Models are defined as follows: model 1: unadjusted model; model 2: adjusted for age, socio-economic status, marital status, menopausal status, hormone replacement therapy, and prevalence of cardiovascular diseases, cancer, or diabetes at recruitment; model 3: model 2 + physical activity, smoking status, alcohol consumption, total energy intake, and Alternate Healthy Eating Index-2010; HR (95% CI), hazard ratio (95% confidence interval).

**Figure 2:**
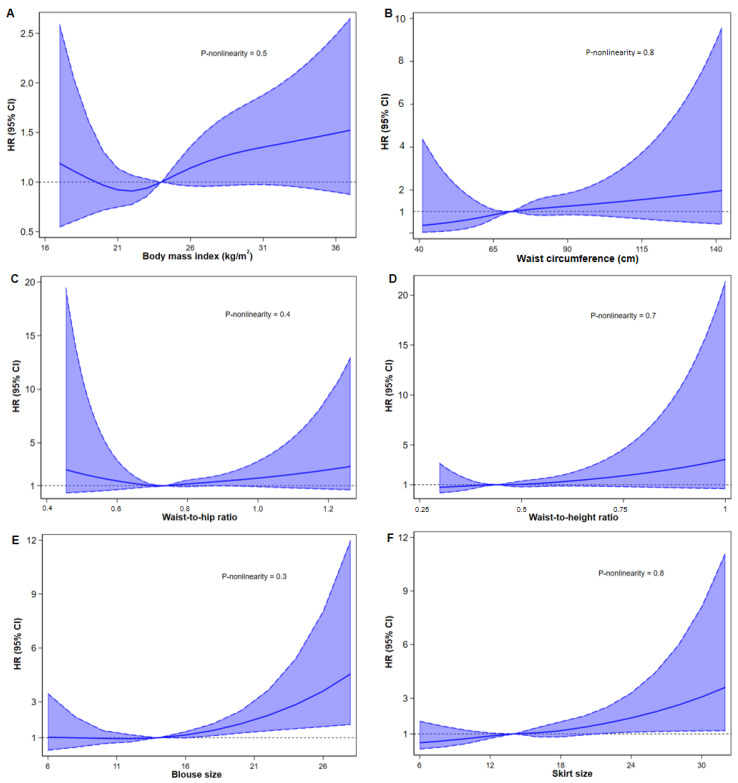
Association between indicators of adiposity with rheumatoid arthritis incidence. Graphs show the dose-response associations between the risk of rheumatoid arthritis and body mass index (**A**), waist circumference (**B**), waist-to-hip ratio (**C**), waist-to-height ratio (**D**), blouse size (**E**), and skirt size (**F**). Estimates were adjusted for age, socio-economic status, marital status, physical activity, smoking status, alcohol consumption, total energy intake, the Alternate Healthy Eating Index-2010, menopausal status, hormone replacement therapy, and the prevalence of CVD, cancer, or diabetes at recruitment. Solid lines are multivariable-adjusted HRs, with shaded areas showing 95% confidence intervals derived from restricted cubic spline regressions with four knots. The dashed horizontal line indicates a reference for no association at an HR 1.0. HR (95% CI), hazard ratio (95% confidence interval).

Figure 1 demonstrates the associations between BMI, WC, WHR, WHtR, blouse size, and skirt size and the incidence of RA. After adjustment for lifestyle factors (model 3), a linear association showed an increased risk of incident RA with BMI (per 2.5 kg/m^2^ increase: HR 1.09 (95% CI: 1.02, 1.16)), WC (per 5 cm increase): 1.06 (1.00, 1.13), WHR (per 0.1 increase: 1.20 (1.01, 1.43)), WHtR (per 0.1 increase: 1.27 (1.05, 1.53)), blouse size (per one size increase: 1.13 (1.04, 1.22)), and skirt size (per one size increase: 1.13 (1.05, 1.22)). Obesity (BMI ≥ 30.0 kg/m^2^) was associated with a 48% higher risk of RA (1.48 (1.02, 2.17)), while abdominal obesity (WC: per 5 cm increase) conferred a 58% higher risk (1.58 (1.10, 2.17)). WHR ≥ 0.85 was associated with a 56% greater risk of RA (1.56 (1.03, 2.36)) and WHtR ≥ 0.6 with a 2.25-fold higher risk (2.25 (1.34, 3.80)). Similarly, skirt size ≥ 20 was linked to a 2.36-fold increased risk of RA (2.36 (1.25, 4.43)). Figure 2 presents non-linear associations, revealing a U-shaped relationship between BMI and RA risk, with the lowest risk around 22 kg/m^2^, though the evidence of association was lacking (P-non-linearity = 0.5). The risk of RA increased monotonically with WC, WHtR, blouse size and skirt size, but there was no evidence of non-linearity in these associations (P-non-linearity > 0.05).

### 3.3. Subgroup Analysis

In subgroup analysis by weight change, we observed effect modification, with WC, WHR, and WHtR showing a linear increase in RA risk only among participants who gained more than 10 kg (P-interaction < 0.05; Supplementary Table S8). Also, menopausal status appeared to influence the associations, with BMI, WC, and WHtR (both continuous and categorical measures) showing an increased risk only in premenopausal women (P-interaction < 0.05; Supplementary Tables S11 and S18). While the risk of RA was significantly higher in individuals younger than 55 years compared to those aged 55 or older, there was no evidence that age modified the associations between obesity indicator and RA risk (Supplementary Table S7). No evidence of effect modification was found according to AHEI-2010 tertiles, physical activity, SES, or smoking status (Supplementary Tables S7–S18).

### 3.4. Sensitivity Analysis

Most of the sensitivity analyses supported the robustness of our main findings. However, when participants with substantial weight gain (>10 kg) were excluded, traditional obesity indicators (BMI, WC, WHR, WHtR) lost their clear associations with the risk of RA, whereas blouse size and skirt size maintained significant associations (blouse size: 1.23 (1.03, 1.46); skirt size: 1.19 (1.04, 1.36); Supplementary Table S19). Additionally, the exclusion of current smokers strengthened the obesity-RA associations: the association of BMI (per 2.5 kg/m^2^ increase) with RA risk increased from 1.09 (1.02, 1.16) to 1.13 (1.06, 1.21); WC (per 5 cm increment) from 1.06 (1.00, 1.13) to 1.11(1.04, 1.19); WHR (per 0.1 increment) from 1.20 (1.01, 1.43) to 1.30 (1.11, 1.53); WHtR (per 0.1 increment) from 1.27 (1.05, 1.53) to 1.42 (1.17, 1.74); blouse size (per one size increment) from 1.13 (1.04, 1.22) to 1.17 (1.07, 1.29); and skirt size (per one size increment) from 1.13 (1.05, 1.22) to 1.17 (1.08, 1.28), indicating that the dominant effect of smoking had previously obscured obesity-related risk (Supplementary Table S19).

## 4. Discussion

### 4.1. Main Findings

This study is the first to simultaneously assess BMI, WC, WHR, WHtR, and clothing size to RA risk in a population of UK women. Our findings support a linear and consistently risk-increasing association between all indicators of obesity-related health and the risk of RA, independent of lifestyle factors, with even stronger positive associations observed in younger women (≤55 years). Participants who did not meet the WHO or NICE guidelines, with a BMI ≥ 30 kg/m^2^, WC > 88 cm, WHR ≥ 0.85, or WHtR ≥ 0.6 had a greater incidence of RA, with WHtR showing the strongest association (a 2.25-fold increased risk). Similarly, a skirt size ≥ 20 was associated with more than a doubling in RA risk. These findings highlight the impact of central obesity on RA risk and emphasise the need for lifestyle interventions to mitigate obesity-related RA risk.

### 4.2. Comparison with Previous Studies

Our findings align with prior prospective cohort studies in US and European populations [18,19,20]. The Danish cohort reported a 46% higher RA risk in individuals with obesity (BMI ≥ 30 kg/m^2^) [20], and our study found a similar 48% increase in middle-aged women, in contrast to the mixed-sex sample in the Danish study. The Nurses’ Health Studies I and II also reported a 37% higher RA risk in overweight US women (BMI: 25.0–<30.0 kg/m^2^), though their confidence intervals were wide [18]. The French E3N cohort reported a 26% higher risk in individuals with obesity (BMI ≥ 30 kg/m^2^), but this was marginally significant [19]. Divergence in risk estimates across studies may be due to differences in case identification: the E3N cohort used self-reported data, whereas our study leveraged the UKWCS-HES linkage for clinically verified diagnoses. A study using the UK General Practice Research Database found no association between obesity and RA risk in over 1 million men and women, potentially due to reliance on physician-recorded BMI data [9]. Consistent with the results from a meta-analysis showing a 13% higher RA risk per 10 cm increase in WC [11], we found a 6% higher risk for every 5 cm increase in WC. This meta-analysis included the Danish cohort [20], but also combined data with a case-control study [21], which may affect the generalisability of the findings.

In this study, our findings permit several important inferences. First, the association of central obesity with RA incidence may be stronger than that of overall obesity as measured by BMI. Indicators such as WC, WHR, WHtR demonstrated stronger associations with RA incidence than BMI. Notably, exceeding the NICE guideline threshold for WHtR (≥0.6) was linked to a substantially greater effect than BMI-defined obesity (≥30 kg/m^2^). Skirt size, which reflects central fat distribution (waist and hips), showed a nearly twofold increase in RA risk when ≥20, whereas blouse size (more reflective of upper body size) showed a weaker association. When excluding participants with substantial weight gain, only blouse size and skirt size retained significant associations with RA risk. This suggests that clothing size may reflect long-term, stable patterns of obesity that are less affected by short-term weight changes. However, clothing size may also be influenced by factors such as fashion trends, socioeconomic status, and cultural norms, which could affect its reliability as a consistent adiposity proxy across populations. These findings suggest that WHtR ≥ 0.6 and skirt size ≥ 20 could serve as practical indicators for identifying individuals at higher risk of RA in primary care settings. Risk assessments and public health guidelines should, therefore, expand beyond BMI to include central obesity informed by clothing size, which better captures adiposity-related disease risk, even in individuals with “normal” BMI. For example, in our cohort, women with WHtR ≥ 0.6 had 2.25-fold higher RA risk, comparable to that associated with smoking. This includes women with normal BMI but elevated WHtR, a high-risk subgroup often missed by traditional screening.

While the HR for BMI of 1.09 corresponds to a relatively small increment (per 2.5 kg/m^2^), the cumulative risk for RA associated with a 10 kg/m^2^ increase in BMI is substantial, with a 41% increased risk. The clinical significance of these small changes becomes more apparent when BMI is categorised.

Second, our sensitivity analyses provide insights into the underlying mechanisms. Excluding current smokers consistently strengthened the associations between all obesity indicators and RA risk, highlighting the possibility that smoking may obscure the role of obesity. This is consistent with findings from the Danish cohort, where central adiposity (assessed by WC or body shape trajectories) predicted RA risk more robustly than BMI among never-smokers [20]. Given the well-established and dominant role of smoking in RA pathogenesis, its exclusion from analyses helps uncover the independent contribution of obesity, likely mediated through distinct metabolic and inflammatory pathways. This finding needs further mechanistic investigation into how adiposity-related inflammation may play a role in RA development independently of smoking-related autoimmune pathways.

Third, after adjusting for lifestyle factors such as smoking, alcohol intake, diet quality, energy intake, and physical activity, the associations between adiposity indicators and RA were attenuated. This suggests that a healthy lifestyle may be associated with a weaker association between obesity and RA. These findings support the rationale for developing preventive strategies targeting modifiable behaviours in individuals with elevated central obesity that could lower their risk of RA.

### 4.3. Possible Mechanistic Explanations

Although excess weight may contribute to joint stress, this mechanism is more pertinent to osteoarthritis [22]. In this study, the stronger associations with central adiposity indicators rather than BMI suggest that systemic inflammation driven by visceral fat is a more plausible pathway linking obesity to RA risk. Excess body fat has been linked to RA risk through chronic low-grade systemic inflammation, insulin resistance, metabolic hormones, gut microbiota alterations [23], and oxidative stress [24]. Enlarged adipocytes secrete pro-inflammatory cytokines such as TNF-α, IL-6, and CRP, while adipose tissue recruits macrophages, further amplifying inflammation. Adipokines like leptin and adiponectin also regulate immune responses; leptin promotes pro-inflammatory T-cell activity [25], while elevated adiponectin exacerbates joint inflammation by activating synovial fibroblasts and promoting cartilage degradation [6]. Additionally, insulin resistance, a well-established consequence of excess abdominal fat, may disrupt immune function and promote systemic inflammation [26]. Other potential mechanisms, such as DNA methylation [27] and telomere shortening [28], could further mediate the link between obesity and RA, independent of direct metabolic changes.

Beyond their biological effects, obesity-related indices also have psychological implications, especially waist-related indicators, influencing individual’s awareness of their body shape and weight status. This “self-perception of obesity” [29] may affect health behaviours, such as dietary choices and physical activity, which in turn impact metabolic health and inflammation.

## 5. Strengths and Limitations

This study benefits from a large sample size, a prospective design, and a long follow-up period, strengthening the reliability of these findings. RA cases were identified using ICD-10 codes from hospital records, minimising misclassification and loss of follow-up. Also, we adjusted for a comprehensive set of confounders, including sociodemographic factors (e.g., age, socioeconomic status), lifestyle factors (physical activity, smoking, alcohol intake, diet quality), reproductive history (menopausal status, hormone replacement therapy), and total energy intake, reducing the risk of residual confounding.

However, residual confounding remains a possibility, particularly from unmeasured factors such as family history and genetic predisposition if they increase both adiposity and RA risk other than through increased adiposity, medication use that increases both adiposity and RA risk other than through adiposity, and specific dietary patterns that independently influence both. Consequently, as in all observational studies, causality cannot be inferred. The absence of inflammatory biomarkers (e.g., CRP, IL-6) limits our ability to investigate the underlying mechanisms linking obesity to RA. Future studies integrating genetic and clinical data may further clarify these associations. Secondly, the generalisability of our findings may be constrained since our cohort comprised predominantly White, middle-aged women in England. This limits the extrapolation of results to men or non-European populations. Third, anthropometric data were based on self-reported measures, which may be subject to reporting bias. However, participants were given standardised instructions, and self-reported anthropometry has been shown to be acceptably accurate in epidemiological research [30]. Any misclassification is likely non-differential, which could attenuate the observed associations rather than exaggerate them. Finally, BMI, WC, WHR, and WHtR are likely to be correlated. While we assessed the association of each measure independently, these indices may jointly influence RA risk. Although we did not formally test interaction effects between obesity indicators, future analyses using combined or interaction models could offer insights into whether combinations of anthropometric traits elevate RA risk.

## 6. Conclusions

The findings of this study suggest the importance of recognising central obesity as a distinct and potentially stronger predictor of RA than BMI. It also highlights the value of alternative, practical indicators such as clothing size in long-term risk assessment. Future research should explore the biological pathways linking adiposity and RA, particularly in non-smoking populations, and evaluate whether lifestyle interventions can modify this relationship.

## Figures and Tables

**Table 1:** Baseline characteristics of 27,968 UKWCS participants stratified by rheumatoid arthritis incidence.

Characteristics	Total	Cases	Non-Cases
Participants (%)	27,968	255 (1.0)	27,713 (99.0)
Age, years (SD)	51.9 (9.2)	53.9 (9.0)	51.9 (9.2)
Degree-level education (%)	7057 (25.2)	48 (18.8)	7009 (25.3)
Socio-economic status (%)			
Professional or managerial	17,753 (63.5)	146 (57.3)	17,607 (63.5)
Intermediate	7735 (27.7)	80 (31.4)	7655 (27.6)
Routine or manual	2480 (8.9)	29 (11.4)	2451 (8.8)
Married (%)	21,207 (75.8)	189 (74.1)	21,018 (75.8)
Anthropometric indices			
Height, cm (SD)	163.7 (6.6)	162.8 (6.8)	163.7 (6.6)
Weight, kg (SD)	65.4 (11.6)	67.2 (14.3)	65.4 (11.5)
Waist circumference, cm (SD)	74.2 (9.6)	76.2 (11.4)	74.2 (9.6)
BMI at year 20, kg/m^2^ (SD)	21.4(2.9)	21.7(3.0)	21.4(2.9)
BMI at recruitment, kg/m^2^ (SD)	24.4 (4.2)	25.4 (5.3)	24.4 (4.2)
Waist-to-hip ratio (SD)	0.8 (0.1)	0.8 (0.1)	0.8 (0.1)
Waist-to-height ratio (SD)	0.5 (0.1)	0.5 (0.1)	0.5 (0.1)
Blouse size (SD)	13.9 (2.9)	14.7 (3.6)	13.9 (2.9)
Skirt size (SD)	14.5 (3.2)	15.4 (3.7)	14.5 (3.2)
Physical activity (METs), hours/week (SD)	16.6 (11.5)	15.4 (9.8)	16.6 (11.5)
Smoking status (%)			
Current	3081 (11.0)	41 (16.1)	3040 (11.0)
Former	8722 (31.2)	89 (34.9)	8633 (31.2)
Never	16,165 (57.8)	125 (49.0)	16,040 (57.9)
Alcohol consumption, grams/week (SD)	9.0 (10.5)	7.1 (8.4)	9.0 (10.5)
Total energy intake, kcal/day (SD)	2334.2 (701.5)	2352.8 (847.3)	2334.1 (700.0)
AHEI-2010 score (SD)	65.6 (11.0)	65.8 (10.1)	65.6 (11.0)
Menopausal status (%)			
Premenopausal	13,571 (48.5)	93 (36.5)	13,571 (49.0)
Postmenopausal	14,397 (51.5)	162 (63.5)	14,397 (52.0)
Hormone replacement therapy (%)	7698 (27.5)	82 (32.2)	7616 (27.5)
Prevalence of CVD, cancer, or diabetes (%)	1908 (6.8)	30 (11.8)	1878 (6.8)

SD standard deviation, BMI body mass index, WC waist circumference, WHR waist-to-hip ratio, WHtR waist-to-height ratio, MET Metabolic Equivalent of Task, AHEI-2010 Alternative Healthy Eating Index-2010, CVD cardiovascular disease. BMI, WC, and WHR were categorised using WHO guidelines, and WHtR was categorised using NICE guidelines.

## Data Availability

The data access policy for the UK Women’s Cohort Study is available on the study’s official website: The UK Women’s Cohort Study (UKWCS), University of Leeds.

## Supplementary Materials

The following supporting information can be downloaded at: https://www.mdpi.com/article/10.3390/nu17091557/s1, Figure S1. Flow chart of UKWCS participants; Table S1. Strengthening the reporting of observational studies in nutritional epidemiology (STROBE-Nut) checklist; Table S2. Obesity group categorisation and definitions; Table S3. Covariates at recruitment and their derivation; Table S4. Proportional Hazards (PH) assumption test for obesity indicators and rheumatoid arthritis risk (in the fully-adjusted model); Table S5. Baseline characteristics of 27,968 UKWCS participants stratified by body mass index, waist circumference, waist-to-hip ratio, and waist-to-height ratio; Table S6. Baseline characteristics of 27,968 UKWCS participants stratified by clothing size; Table S7. Associations between obesity indicators and rheumatoid arthritis incidence in UK Women’s Cohort Study participants, stratified by age; Table S8. Associations between adiposity indicators and rheumatoid arthritis incidence in UK Women’s Cohort Study participants, stratified by weight change; Table S9. Associations between obesity indicators and rheumatoid arthritis incidence in UK Women’s Cohort Study participants, stratified by AHEI-2010; Table S10. Associations between obesity indicators and rheumatoid arthritis incidence in UK Women’s Cohort Study participants, stratified by physical activity; Table S11. Associations between obesity indicators and rheumatoid arthritis incidence in UK Women’s Cohort Study participants, stratified by menopausal status; Table S12. Associations between obesity indicators and rheumatoid arthritis incidence in UK Women’s Cohort Study participants, stratified by smoking status; Table S13. Associations between obesity indicators and rheumatoid arthritis incidence in UK Women’s Cohort Study participants, stratified by age; Table S14. Associations between obesity indicators and rheumatoid arthritis incidence in UK Women’s Cohort Study participants, stratified by weight change; Table S15. Associations between obesity indicators and rheumatoid arthritis incidence in UK Women’s Cohort Study participants, stratified by AHEI-2010; Table S16. Associations between obesity indicators and rheumatoid arthritis incidence in UK Women’s Cohort Study participants, stratified by physical activity; Table S17. Associations between obesity indicators and rheumatoid arthritis incidence in UK Women’s Cohort Study participants, stratified by smoking status; Table S18. Associations between obesity indicators and rheumatoid arthritis incidence in UK Women’s Cohort Study participants, stratified by menopausal status; Table S19. Risk of rheumatoid arthritis by obesity indicators with varying restrictions in the UKWCS; Supplementary Methods.

## References

[1] (2023). Global, regional, and national burden of rheumatoid arthritis, 1990–2020, and projections to 2050: A systematic analysis of the Global Burden of Disease Study 2021. Lancet Rheumatol..

[2] Casale R., Atzeni F., Bazzichi L., Beretta G., Costantini E., Sacerdote P., Tassorelli C. (2021). Pain in Women: A Perspective Review on a Relevant Clinical Issue that Deserves Prioritization. Pain Ther..

[3] (2022). Digital N: Health Survey for England 2021: Overweight and Obesity (Part 2). https://digital.nhs.uk/data-and-information/publications/statistical/health-survey-for-england/2021/part-2-overweight-and-obesity.

[4] Rubino F., Cummings D.E., Eckel R.H., Cohen R.V., Wilding J.P.H., Brown W.A., Stanford F.C., Batterham R.L., Farooqi I.S., Farpour-Lambert N.J. (2025). Definition and diagnostic criteria of clinical obesity. Lancet. Diabetes Endocrinol..

[5] Powell-Wiley T.M., Poirier P., Burke L.E., Després J.P., Gordon-Larsen P., Lavie C.J., Lear S.A., Ndumele C.E., Neeland I.J., Sanders P. (2021). Obesity and Cardiovascular Disease: A Scientific Statement From the American Heart Association. Circulation.

[6] NICE (2022). Keep the Size of Your Waist to Less Than Half of Your Height, NICE Recommends. https://www.nice.org.uk/news/articles/keep-the-size-of-your-waist-to-less-than-half-of-your-height-nice--recommends.

[7] Zhou Y., Sun M. (2018). A meta-analysis of the relationship between body mass index and risk of rheumatoid arthritis. EXCLI J..

[8] Qin B., Yang M., Fu H., Ma N., Wei T., Tang Q., Hu Z., Liang Y., Yang Z., Zhong R. (2015). Body mass index and the risk of rheumatoid arthritis: A systematic review and dose-response meta-analysis. Arthritis Res Ther..

[9] Rodríguez L.A., Tolosa L.B., Ruigómez A., Johansson S., Wallander M.A. (2009). Rheumatoid arthritis in UK primary care: Incidence and prior morbidity. Scand. J. Rheumatol..

[10] Lee J.S., Oh J.S., Kim S., Kim Y.J., Hong S., Kim Y.G., Lee C.K., Yoo B. (2024). The association of obesity and the risk of rheumatoid arthritis according to abdominal obesity status: A nationwide population-based study in Korea. Rheumatol. Int..

[11] Ohno T., Aune D., Heath A.K. (2020). Adiposity and the risk of rheumatoid arthritis: A systematic review and meta-analysis of cohort studies. Sci. Rep..

[12] Marchand N.E., Sparks J.A., Tedeschi S.K., Malspeis S., Costenbader K.H., Karlson E.W., Lu B. (2021). Abdominal Obesity in Comparison with General Obesity and Risk of Developing Rheumatoid Arthritis in Women. J. Rheumatol..

[13] Lachat C., Hawwash D., Ocké M.C., Berg C., Forsum E., Hörnell A., Larsson C., Sonestedt E., Wirfält E., Åkesson A. (2016). Strengthening the Reporting of Observational Studies in Epidemiology-Nutritional Epidemiology (STROBE-nut): An Extension of the STROBE Statement. PLoS Med..

[14] Cade J.E., Burley V.J., Alwan N., Hutchinson J. (2017). Cohort profile: The UK Women’s cohort study (UKWCS). Int. J. Epidemiol..

[15] Moy F.M., Greenwood D.C., Cade J.E. (2018). Associations of clothing size, adiposity and weight change with risk of postmenopausal breast cancer in the UK Women’s Cohort Study (UKWCS). BMJ Open.

[16] Threapleton D.E., Greenwood D.C., Burley V.J., Aldwairji M., Cade J.E. (2013). Dietary fibre and cardiovascular disease mortality in the UK Women’s Cohort Study. Eur. J. Epidemiol..

[17] Wang T., Heianza Y., Sun D., Huang T., Ma W., Rimm E.B., Manson J.E., Hu F.B., Willett W.C., Qi L. (2018). Improving adherence to healthy dietary patterns, genetic risk, and long term weight gain: Gene-diet interaction analysis in two prospective cohort studies. BMJ.

[18] Lu B., Hiraki L.T., Sparks J.A., Malspeis S., Chen C.Y., Awosogba J.A., Arkema E.V., Costenbader K.H., Karlson E.W. (2014). Being overweight or obese and risk of developing rheumatoid arthritis among women: A prospective cohort study. Ann. Rheum. Dis..

[19] Salliot C., Nguyen Y., Mariette X., Boutron-Ruault M.C., Seror R. (2022). Anthropometric Measures and Risk of Rheumatoid Arthritis in the French E3N Cohort Study. Nutrients.

[20] Linauskas A., Overvad K., Symmons D., Johansen M.B., Stengaard-Pedersen K., de Thurah A. (2019). Body Fat Percentage, Waist Circumference, and Obesity As Risk Factors for Rheumatoid Arthritis: A Danish Cohort Study. Arthritis Care Res..

[21] Ljung L., Rantapää-Dahlqvist S. (2016). Abdominal obesity, gender and the risk of rheumatoid arthritis—A nested case-control study. Arthritis Res. Ther..

[22] Gao T., Chen Z.Y., Li T., Lin X., Hu H.G., Tang J.D., Wu C. (2025). Association between body roundness index and osteoarthritis/rheumatoid arthritis: A cross-sectional study. Sci. Rep..

[23] Zaiss M.M., Joyce Wu H.-J., Mauro D., Schett G., Ciccia F.J.N.R.R. (2021). The gut–joint axis in rheumatoid arthritis. Nat. Rev. Rheumatol..

[24] Rohm T.V., Meier D.T., Olefsky J.M., Donath M.Y. (2022). Inflammation in obesity, diabetes, and related disorders. Immunity.

[25] Kiernan K., MacIver N.J. (2020). The Role of the Adipokine Leptin in Immune Cell Function in Health and Disease. Front. Immunol..

[26] Nicolau J., Lequerré T., Bacquet H., Vittecoq O. (2017). Rheumatoid arthritis, insulin resistance, and diabetes. Jt. Bone Spine.

[27] Payet M., Dargai F., Gasque P., Guillot X. (2021). Epigenetic regulation (including micro-RNAs, DNA methylation and histone modifications) of rheumatoid arthritis: A systematic review. Int. J. Mol. Sci..

[28] Costenbader K.H., Prescott J., Zee R.Y., De Vivo I. (2011). Immunosenescence and rheumatoid arthritis: Does telomere shortening predict impending disease?. Autoimmun. Rev..

[29] Robinson E., Haynes A., Sutin A., Daly M. (2020). Practice: Self-perception of overweight and obesity: A review of mental and physical health outcomes. Obes. Sci. Pract..

[30] Wright F.L., Green J., Reeves G., Beral V., Cairns B.J. (2015). Validity over time of self-reported anthropometric variables during follow-up of a large cohort of UK women. BMC Med. Res. Methodol..

