# Supplementary material for: Measures of Adiposity and Risk of Rheumatoid Arthritis in Middle-Aged UK Women: A Prospective Cohort Study
Source: Nutrients. 2025 Apr 30;17(9):1557. doi: 10.3390/nu17091557 (PMC12074165; doi:10.3390/nu17091557)
Supplement: Supplementary file 1 [file nutrients-17-01557-s001.zip › nutrients-3604732-supplementary.pdf]

Measures of Adiposity and Risk of Rheumatoid Arthritis in Middle-aged UK Women: A Prospective Cohort Study

Yuanyuan Dong[1*], Darren C. Greenwood[2], Laura J. Hardie[3], Janet E. Cade[1]

Contents

Supplemental Figures:....4
Figure S1. Flow chart of UKWCS participants....4
Supplemental Tables:....5
Table S1. Strengthening the reporting of observational studies in nutritional epidemiology (STROBE-Nut) checklist....5
Table S2. Obesity group categorisation and definitions. 9
Table S3. Covariates at recruitment and their derivation. 10
Table S4. The proportional hazards assumption test for obesity indicators and rheumatoid arthritis risk (in the fully-adjusted model)....13
Table S5. Baseline characteristics of 27,968 UKWCS participants stratified by body mass index, waist circumference, waist-to-hip ratio, and waist-to-height ratio....14
Table S6. Baseline characteristics of 27,968 UKWCS participants stratified by clothing size....16
Table S7. Associations between obesity indicators and rheumatoid arthritis incidence in UK Women's Cohort Study participants, stratified by age. 18
Table S8. Associations between adiposity indicators and rheumatoid arthritis incidence in UK Women's Cohort Study participants, stratified by weight change. . 19
Table S9. Associations between obesity indicators and rheumatoid arthritis incidence in UK Women's Cohort Study participants, stratified by AHEI-2010....20
Table S10. Associations between obesity indicators and rheumatoid arthritis incidence in UK Women's Cohort Study participants, stratified by physical activity.21
Table S11. Associations between obesity indicators and rheumatoid arthritis incidence in UK Women's Cohort Study participants, stratified by menopausal status. 22
Table S12. Associations between obesity indicators and rheumatoid arthritis incidence in UK Women's Cohort Study participants, stratified by smoking status. 23
Table S13. Associations between obesity indicators and rheumatoid arthritis incidence in UK Women's Cohort Study participants, stratified by age....24
Table S14. Associations between obesity indicators and rheumatoid arthritis incidence in UK Women's Cohort Study participants, stratified by weight change. . 26
Table S15. Associations between obesity indicators and rheumatoid arthritis incidence in UK Women's Cohort Study participants, stratified by AHEI-2010. 28
Table S16. Associations between obesity indicators and rheumatoid arthritis incidence in UK Women's Cohort Study participants, stratified by physical activity.30
Table S17. Associations between obesity indicators and rheumatoid arthritis incidence in UK Women's Cohort Study participants, stratified by smoking status. 32
Table S18. Associations between obesity indicators and rheumatoid arthritis incidence in UK Women's Cohort Study participants, stratified by menopausal status. 34
Table S19. Risk of rheumatoid arthritis by obesity indicators with varying restrictions in the UKWCS....36

Supplementary methods: 46

Supplemental Figures:

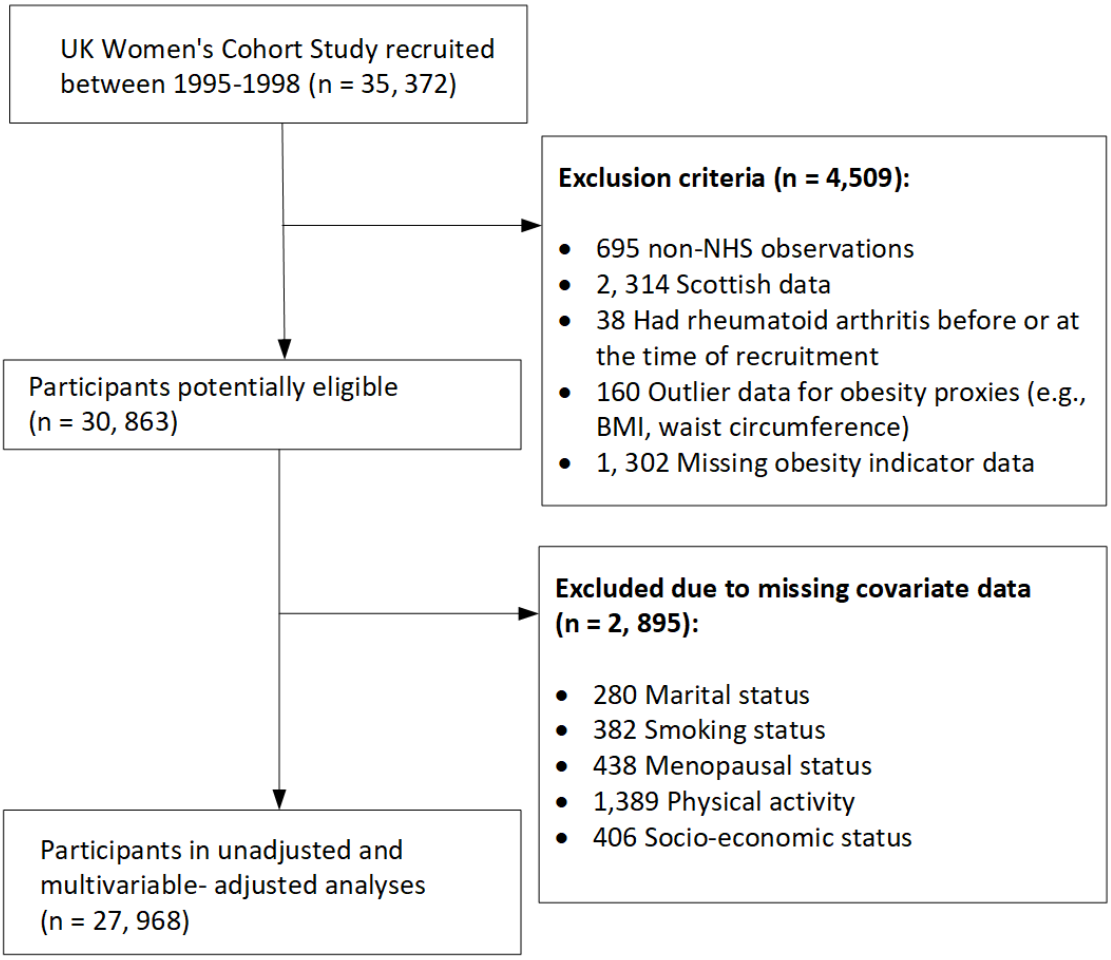

Figure S1. Flow chart of UKWCS participants.

Supplemental Tables:

Table S1. Strengthening the reporting of observational studies in nutritional epidemiology (STROBE-Nut) checklist.

**Topic**	**Item number**	**Recommendation**	**Page (line number)**
Title and abstract	1	Indicate the study's design with a commonly used term in the title or the abstract	1(1-4)
		Provide in the abstract an informative and balanced summary of what was done and what was found	1(12-36)
Introduction			
Background/rationale	2	Explain the scientific background and rationale for the investigation being reported	1(38-42), 2(43-77)
Objectives	3	State specific objectives, including any prespecified hypotheses	2(68-77)
Methods			
Study design	4	Present key elements of study design early in the manuscript	3(84-100)
Setting	5	Describe the setting, locations, and relevant dates, including periods of recruitment, exposure, follow-up, and data collection	3(84-100)
Participants	6	Cohort study - give the eligibility criteria, and the sources and methods of selection of participants; describe methods of follow-up	4(139-142), Supplementary Table A1.
		Cohort study - for matched studies, give matching criteria and number of exposed and unexposed Case-control study - for matched studies, give matching criteria and the number of controls per case	N/A
Variables	7	Clearly define all outcomes, exposures, predictors, potential confounders, and effect modifiers; give diagnostic criteria, if applicable	3(101-125), 4(125-147)<br>Table A1, Table

			A5, A6
Data sources/measurement	8*	For each variable of interest, give sources of data and details of methods of assessment (measurement); describe comparability of assessment methods if there is more than one group	3(80-125), 4(126-147) Table A1, Figure A1, Table A5 and A6
Bias	9	Describe any efforts to address potential sources of bias	4(151-174), 5(175-188)
Study size	10	Explain how the study size was arrived at	5(190-193), Figure A1
Quantitative variables	11	Explain how quantitative variables were handled in the analyses; if applicable, describe which groupings were chosen and why	4(151-162)
Statistical methods	12	Describe all statistical methods, including those used to control for confounding	4(143-170) 5(171-183)
		Describe any methods used to examine subgroups and interactions	4(172-174), 5(175-180)
		Explain how missing data were addressed	5(185-188)
		Cohort study - if applicable, explain how loss to follow-up was addressed	N/A
		Describe any sensitivity analyses	5(182-188)
Results			
Participants	13*	Report numbers of individuals at each stage of study - e.g., numbers potentially eligible, examined for eligibility, confirmed eligible, included in the study, completing follow-up, and analyzed	5(190-193)
		Give reasons for nonparticipation at each stage Consider use of a flow diagram	Figure A1
Descriptive data	14*	Give characteristics of study participants (e.g., demographic, clinical, social) and information on	5(190-221)

		exposures and potential confounders	
		Indicate number of participants with missing data for each variable of interest Cohort study - summarize follow-up time (e.g., average and total amount)	5(190-221)
Outcome data	15*	Cohort study - report numbers of outcome events or summary measures over time	5(184-214)<br>Table 1, Table A5 and A6
Main results	16	Give unadjusted estimates and, if applicable, confounder-adjusted estimates and their precision (e.g., 95% confidence interval); make clear which confounders were adjusted for and why they were included Report category boundaries when continuous variables were categorized	Figure 1,<br>Figure 2
		If relevant, consider translating estimates of relative risk into absolute risk for a meaningful time period	N/A
Other analyses	17	Report other analyses done - e.g., analyses of subgroups and interactions, and sensitivity analyses	6(239-264), Table A7-A19
Discussion			
Key results	18	Summarize key results with reference to study objectives	10(292-302)
Limitations	19	Discuss limitations of the study, taking into account sources of potential bias or imprecision; discuss both direction and magnitude of any potential bias	12(394-414)
Interpretation	20	Give a cautious overall interpretation of results considering objectives, limitations, multiplicity of analyses, results from similar studies, and other relevant evidence	11(303-337),12(338-383)
Generalizability	21	Discuss the generalizability (external validity) of the study results	13(416-421)
Other information			
Funding	22	Give the source of funding and the role of the funders for the present study and, if applicable, for the original study on which the present article is based	14(457-458)

Supplementary Materials

Table S2. Adiposity group categorisation and definitions.

Classification Systems	Category	Definition
**BMI**	Underweight	BMI < 18.5 kg/m²
	Normal weight	BMI 18.5 - < 25 kg/m²
	Overweight	BMI 25.0 - < 30 kg/m²
	Obese	BMI ≥30.0 kg/m²
**WC**	No abdominal obesity	WC ≤88 cm
	Abdominal obesity	WC > 88 cm
**WHR**	Normal	WHR: < 0.8
		WHR: 0.8-0.85
	Elevated	WHR ≥ 0.85
**WHtR**	Normal	WHtR < 0.5
	Overweight	WHtR 0.5- < 0.6
	Obese	WHtR ≥ 0.6

BMI, WC, and WHR categories are defined by World Health Organization (WHO) criteria[1]. WHtR categories are defined by NICE criteria[2]. BMI: body mass index. WC: waist circumference. WHR: waist-to-hip ratio. WHtR: waist-to-height ratio.

[2] Ashwell M, Mayhew L, Richardson J, Rickayzen B. Waist-to-height ratio is more predictive of years of life lost than body mass index. PLoS One. 2014 Sep 8;9(9):e103483. doi: 10.1371/journal.pone.0103483.

Table S3. Covariates at recruitment and their derivation.

**Covariate**	**How the variable was derived**
**Socio-demographic variables**	
Age	Calculated as year differences between date of birth and date of recruitment and was considered a continuous variable in adjustment sets.
Socio-economic status	Participants were asked about their occupation. Options were 'never had paid job', 'managers and administrators', 'professional', 'technical and associate professional', 'clerical and secretarial', 'craft and skilled', 'personal and protective', 'sales', 'plant and machine operatives', or 'other'. We condensed these options into 'routine/manual', 'intermediate', or 'managerial/professional'.
Education level	Participants were asked what their highest educational qualification was. Options were 'no qualifications', 'O level', 'A level', 'degree', or 'missing'.
Marriage status	Participants were asked 'what is your marital status?' with options of 'married or living as married', 'divorced', 'widowed', 'single', or 'separated'. We combined 'divorced' and 'separated' together, and 'widowed' and 'single' together.
Weight change	Participants were asked about weight at age 20 and current weight from the baseline survey. We categorised weight change as a categorical variable: 'weight loss' (< -2.5 kg), 'weight gain' (moderate: 2.5 to 10 kg; large: > 10 kg), and 'stable weight' ($\pm$2.5 kg).
**Lifestyle and other variables**	
Physical activity	Participants reported the type (e.g., walking, cycling, housework, gardening) and duration of physical activity per week. Physical activity levels were calculated using METs and categorized as low activity (< 600 MET-min/week), moderate activity (600-1500 MET-min/week), and high activity (> 1500 MET-min/week).

Smoking status	Participants were asked to describe their smoking habit as 'smoke daily', 'smoke occasionally', 'ex-smoker', or 'never'. We combined daily and occasional smokers into 'smokers', and kept 'ex-smoker' and 'never smoked' the same.
Alcohol consumption	Participants were asked how often they drink alcohol. Options were '> 1/week', '1/week', '< 1/week', or 'never'. This was computed as grams per week.
Energy intake	Participants were asked how often they eat the specific food item and completed a 217-item FFQs. Portion sizes were assigned based on McCance and Widdowson's Composition of Foods database, which provides standard UK portion sizes. Each food item in the FFQ was linked to nutrient composition tables to calculate total energy intake. This was computed as kcal per day.
AHEI-2010	AHEI-2010 was used to assess diet quality based on adherence to dietary recommendations, consisting of 11 food components. It was calculated using food intake data from a validated 217-item FFQs. The total AHEI-2010 score was obtained by summing across 11 food components (vegetables, fruits, whole grains, sugar-sweetened beverages and fruit juice, nuts and legumes, red and processed meat, trans fat, polyunsaturated fatty acids, long-chain omega-3 fats, sodium, alcohol), and participants were categorised into tertiles based on their AHEI-2010 scores.
Menopausal status	Categorised participants as pre-menopausal or post-menopausal. Criteria for postmenopausal was: age > 55 years, both ovaries removed, currently on hormone replacement therapy, or no periods in the last 12 months.
Hormone replacement therapy	Participants were asked 'have you ever used hormone replacement therapy?' and 'are you using HRT now?' – based on these yes or no answers, we categorised hormone replacement therapy use as 'current', 'ex-user', and 'never'.
Prevalence of CVD, cancer, or diabetes	Participants were asked 'Have you ever had a XX' including cancer, stroke, and diabetes; - based on these yes

	or no answers, we categorised prevalence of CVD, cancer, or diabetes as 'Yes' and 'No'.

METS: Metabolic Equivalent of Task, FFQ: food frequency questionnaire, AHEI-2010:Alternate Healthy Eating Index-2010, CVD: cardiovascular disease.

Table S4. The proportional hazards assumption test for obesity indicators and rheumatoid arthritis risk (in the fully-adjusted model)

Obesity Indicator	PH Test (P-Value)
**BMI**	
- Continuous (per 1 kg/m^2)	0.18
- Categorical (normal, overweight, obese)	0.16
**WC**	
- Continuous (per 5 cm increment)	0.21
- Categorical (≤ 88 cm vs. > 88 cm)	0.28
**WHR categories (≤ 0.85 vs. >0.85)**	0.18
**WHtR categories (< 0.5, 0.5–0.6, ≥ 0.6)**	0.20
**Blouse sizes**	
- Continuous (per 2-sizes increment)	0.26
- Categorical (8-10, 12-14, ≥ 16)	0.38
**Skirt sizes**	
- Continuous (per 2-sizes increment)	0.24
- Categorical (8-10, 12-14, ≥ 16)	0.46

The PH assumption was tested using Schoenfeld residuals. A P-value < 0.05 indicates a violation of the PH assumption. PH: proportional hazards; BMI: body mass index; WC: waist circumference; WHR: waist-to-hip ratio; WHtR: waist-to-height ratio.

Table S5. Baseline characteristics of 27,968 UKWCS participants stratified by body mass index, waist circumference, waist-to-hip ratio, and waist-to-height ratio.

Characteristics	BMI (kg/m²)			WC (cm)		WHR			WHtR		
	18.5 -< 25.0	25.0-< 30.0	≥ 30.0	≤ 88	> 88	≤ 0.80	0.80-0.85	> 0.85	< 0.5	0.5 -< 0.6	≥ 0.6
**Participants**	17558	7035	2679	25307	2406	22848	3186	1934	22212	4764	737
**Cases(%)**	141()	74()	36()	218()	37()	189()	38()	28()	182()	57()	16()
**Age, years (SD)**	51.0(9.1)	53.7(9.1)	53.7(9.1)	51.5(9.0)	55.8(9.2)	51.4(9.0)	53.6(9.7)	55.0(10.0)	51.0(8.9)	55.4(9.5)	56.2(9.3)
**Degree-level education(%)**	4957(28.2)	1423(20.2)	486(18.1)	6639(26.2)	418(17.4)	5957(26.1)	738(23.2)	362(18.7)	6045(27.2)	906(19.0)	106(14.4)
**Socio-economic status (%)**											
Professional or managerial	11449(65.2)	4321(61.4)	1618(60.4)	16291(64.4)	1462(60.8)	14550(63.7)	2022(63.5)	1181(61.1)	14409(64.9)	2911(61.1)	433(58.8)
Intermediate	4797(27.3)	2055(29.2)	739(27.6)	7016(27.7)	719(29.9)	6335(27.7)	849(26.6)	551(28.5)	6114(27.5)	1407(29.5)	214(29.0)
Routine or manual	1453(12.7)	659(9.4)	322(12.0)	2218(8.8)	262(10.9)	1963(8.6)	315(9.9)	202(10.4)	1871(8.4)	503(10.6)	106(14.4)
**Married (%)**	13489(76.8)	5357(76.1)	1989(74.2)	19454(76.9)	1753(72.9)	17480(76.5)	2349(73.7)	1378(71.3)	17127(77.1)	3555(74.6)	525(71.2)
**Physical activity (METS), h/week (SD)**	16.8(11.4)	16.1(11.0)	15.8(12.9)	16.6(11.3)	16.1(13.1)	16.6(11.3)	16.6(12.0)	16.5(12.7)	16.7(11.3)	16.2(12.1)	15.8(12.6)

**Smoking status (%)**											
Current	2022(11.5)	678(9.6)	275(10.3)	2849(11.3)	232(9.6)	2501(10.9)	367(11.5)	213(11.0)	2550(11.5)	459(9.6)	72(9.8)
Former	5223(29.7)	2371(33.7)	1007(37.6)	7788(30.8)	934(38.8)	6886(30.1)	1105(34.7)	731(37.8)	6673(30.0)	1755(36.9)	294(39.9)
Never	10454(59.5)	3986(56.7)	1397(52.1)	14888(58.8)	1277(53.1)	13461(58.9)	1714(53.8)	990(51.2)	13171(59.3)	2607(54.7)	387(52.5)
**Alcohol consumption, g/week (SD)**	9.4(10.4)	8.7(10.5)	6.8(10.4)	9.1(10.4)	7.5(11.2)	9.0(10.2)	8.8(11.2)	8.7(12.1)	9.2(10.3)	8.1(10.7)	6.3(12.2)
**Total energy intake, kcal/day (SD)**	2348(696.6)	2308(689.2)	2314(746.9)	2332(698.7)	2350(730.0)	2331(699.7)	2355(697.8)	2334(728.3)	2333(698.3)	2336(703.3)	2348(781.5)
**AHEI-2010 score (SD)**	66.4(11.9)	66.4(10.8)	63.3(10.8)	65.8(11.0)	63.4(10.8)	65.9(10.9)	64.9(10.9)	63.3(1.2)	66.1(11.0)	64.0(10.9)	62.9(10.8)
**Menopausal status (%)**											
Pre-menopausal	9346(53.2)	2862(40.7)	1055(39.4)	12782(50.5)	789(32.8)	11511(50.4)	1358(42.6)	702(36.3)	11690(52.6)	1652(34.7)	229(31.1)
Postmenopausal	8353(47.6)	4173(59.3)	1624(60.6)	12743(50.4)	1654(68.7)	11337(49.6)	1828(57.4)	1232(63.7)	10704(48.2)	3169(66.5)	524(71.1)
**Hormone replacement therapy (%)**	4516(25.7)	2258(32.1)	817(30.5)	6996(27.6)	702(29.2)	6285(27.5)	873(27.4)	540(27.9)	5995(27.0)	1496(31.4)	207(28.1)
**Prevalence of CVD, cancer, or diabetes (%)**	1027(5.8)	527(7.5)	309(11.5)	1619(6.4)	289(12.0)	1421(6.2)	295(9.3)	192(9.9)	1358(6.1)	435(9.1)	115(15.6)

SD standard deviation, BMI body mass index, WC waist circumference, WHR waist-to-hip ratio, WHtR waist-to-height ratio, MET Metabolic Equivalent of Task, AHEI-2010 Alternative Healthy Eating Index-2010, CVD cardiovascular diseases.

Table S6. Baseline characteristics of 27,968 UKWCS participants stratified by clothing size.

Characteristics	Blouse size						Skirt size					
	≤ 10	12	14	16	18	≥ 20	≤ 10	12	14	16	18	≥ 20
**Participants**	3719	8222	7670	4667	2063	1467	2809	6774	7901	5476	2750	2018
**Cases(%)**	29(0.8)	66(0.8)	57(0.7)	53(1.1)	26(1.3)	24(1.6)	15(0.5)	51(0.8)	68(0.9)	63(1.2)	24(0.9)	24(1.2)
**Age, years (SD)**	48.2(7.9)	49.8(8.6)	52.4(9.1)	54.6(9.3)	56.1(9.2)	55.9(9.2)	47.1(7.5)	48.9(8.0)	51.7(9.0)	54.4(9.2)	56.3(9.3)	56.9(9.3)
**Degree-level education(%)**	1173(31.5)	2375(28.9)	1868(24.4)	1017(21.8)	377(18.3)	247(16.8)	922(32.8)	2030(30.0)	1989(25.2)	1231(22.5)	533(19.4)	352(17.4)
**Socio-economic status (%)**												
Professional or managerial	2475(66.6)	5348(65.0)	4828(62.9)	2931(62.8)	1286(62.3)	885(60.3)	1914(68.1)	4441(65.6)	5027(63.6)	3412(62.3)	1742(63.3)	1217(60.3)
Intermediate	981(26.4)	2220(27.0)	2196(28.6)	1348(28.9)	569(27.6)	421(28.7)	699(24.9)	1850(27.3)	2228(28.2)	1607(29.3)	768(27.9)	583(28.9)
Routine or manual	263(7.1)	654(79.5)	703(91.7)	441(9.4)	234(11.3)	185(12.6)	196(7.0)	534(7.9)	714(9.0)	520(9.5)	264(9.6)	252(12.5)
**Married (%)**	2846(76.5)	6339(77.1)	5934(77.4)	3511(75.2)	1538(74.6)	1039(70.8)	2112(75.2)	5261(77.7)	6166(78.0)	4204(76.8)	2041(74.2)	1423(70.5)
**Physical activity (METs), h/week (SD)**	17.1(11.5)	17.0(11.4)	16.4(11.2)	16.2(11.0)	16.1(12.6)	15.8(12.6)	17.7(12.0)	17.1(11.6)	16.7(11.2)	16.0(10.9)	15.9(12.0)	15.4(12.0)

**Smoking status (%)**												
Current	483(13.0)	928(11.3)	810(10.6)	497(10.6)	201(9.7)	162(11.0)	424(15.1)	801(11.8)	859(10.9)	558(10.2)	231(8.4)	208(10.3)
Former	942(25.3)	2419(29.4)	2448(31.9)	1588(34.0)	767(37.2)	558(38.0)	714(25.4)	2013(29.7)	2471(31.3)	1813(33.1)	952(34.6)	759(37.6)
Never	2294(61.7)	4875(59.3)	4469(58.3)	2635(56.5)	1121(54.3)	771(52.6)	1671(59.5)	4011(59.2)	4639(58.7)	3168(57.9)	1591(57.9)	1085(53.8)
**Alcohol consumption, g/week (SD)**	9.2(9.9)	9.5(10.3)	9.3(10.7)	8.7(10.7)	7.9(10.7)	5.9(9.3)	9.6(10.3)	9.7(10.5)	9.3(10.5)	8.8(10.7)	7.8(10.2)	6.2(9.1)
**Total energy intake, kcal/day (SD)**	2288(700.0)	2335(693.0)	2345(689.1)	2349(704.6)	2321(739.9)	2356(744.7)	2287(714.8)	2328(697.8)	2339(687.5)	2350(691.1)	2334(723.2)	2353(744.0)
**AHEI-2010 score (SD)**	66.8(11.0)	66.4(11.0)	65.7(10.9)	64.8(10.9)	63.8(10.9)	62.8(10.9)	67.0(11.2)	66.8(11.0)	65.7(10.9)	64.9(10.8)	64.4(10.8)	63.2(10.8)
**Menopausal status (%)**												
Premenopausal	2412(64.9)	4752(57.8)	3619(47.2)	1703(36.5)	638(30.9)	447(30.5)	1925(68.5)	4190(61.9)	3979(50.4)	2076(37.9)	845(30.7)	556(27.6)
Postmenopausal	1307(35.1)	3470(42.2)	4108(53.6)	3017(64.6)	1451(70.3)	1044(71.2)	884(31.5)	2635(38.9)	3990(50.5)	3463(63.2)	1920(69.8)	1496(74.1)
**Hormone replacement therapy (%)**	791(21.3)	2087(25.4)	2221(29.0)	1513(32.4)	671(32.5)	415(28.3)	564(20.1)	1627(24.0)	2308(29.2)	1747(31.9)	856(31.1)	596(29.5)
**Prevalence of CVD, cancer, or diabetes (%)**	184(4.9)	458(5.6)	499(6.5)	340(9.7)	205(9.9)	222(15.1)	145(5.2)	331(4.9)	498(6.3)	396(7.2)	251(9.1)	287(14.2)

SD standard deviation, MET Metabolic Equivalent of Task, AHEI-2010 Alternative Healthy Eating Index-2010, CVD cardiovascular diseases.

Table S7. Associations between adiposity indicators and rheumatoid arthritis incidence in UK Women's Cohort Study participants, stratified by age.

Obesity Indicator	Age (years)		*P*-interaction
	< 55<br>(142/18252)	≥ 55<br>(113/9716)	
**BMI (per 2.5 kg/m² increment)**	1.16(1.08, 1.24)	1.04(0.92, 1.17)	0.1
**WC (per 5 cm increment)**	1.14(1.05, 1.23)	1.02(0.92, 1.12)	0.1
**WHR (per 0.1 increment)**	1.32(1.05, 1.64)	1.12(0.86, 1.47)	0.4
**WHtR (per 0.1 increment)**	1.54(1.26, 1.88)	1.11(0.81, 1.51)	0.1
**Blouse size (per one-size increase)**	1.21(1.09, 1.34)	1.09(0.95, 1.25)	0.2
**Skirt size (per one-size increase)**	1.21(1.10, 1.33)	1.10(0.98, 1.24)	0.2

Models were adjusted for socio-economic status, marital status, menopausal status, hormone replacement therapy, and prevalence of CVD, cancer, or diabetes at recruitment, physical activity, smoking status, alcohol consumption, total energy intake, and Alternate Healthy Eating Index -2010; HR (95% CI), hazard ratio (95% confidence interval).

Table S8. Associations between adiposity indicators and rheumatoid arthritis incidence in UK Women's Cohort Study participants, stratified by weight change.

Adiposity Indicator	Weight change (kg)				*P*-interaction
	Weight loss (< -2.5) (31/4147)	Stable weight (±2.5) (26/2909)	Weight gain, moderate (2.5-< 10) (98/10657)	Weight gain, large (≥ 10) (108/10255)	
**BMI (per 2.5 kg/m^2 increment)**	1.06(0.73, 1.56)	1.15(0.83, 1.59)	1.02(0.88, 1.19)	1.14(1.03, 1.26)	0.8
**WC (per 5 cm increment)**	0.70(0.50, 0.97)	1.11(0.89, 1.38)	0.95(0.81, 1.11)	**1.15(1.06, 1.24)**	**0.03**
**WHR (per 0.1 increment)**	0.60(0.28, 1.28)	0.98(0.57, 170)	0.83(0.56, 1.24)	**1.46(1.24, 1.74)**	**0.01**
**WHtR (per 0.1 increment)**	0.49(0.21, 1.19)	1.39(0.67, 2.87)	0.92(0.59, 1.44)	**1.57(1.27, 1.94)**	**0.05**
**Blouse size (per one-size increase)**	1.02(0.58, 1.81)	1.43(1.06, 1.93)	1.21(0.97, 1.52)	1.11(0.98, 1.26)	0.4
**Skirt size (per one-size increase)**	1.12(0.79, 1.58)	1.31(1.07, 1.61)	1.23(1.04, 1.44)	1.11(0.99, 1.26)	0.4

Models were adjusted for age, socio-economic status, marital status, menopausal status, hormone replacement therapy, and prevalence of CVD, cancer, or diabetes at recruitment, physical activity, smoking status, alcohol consumption, total energy intake, and Alternate Healthy Eating Index -2010; HR (95% CI), hazard ratio (95% confidence interval).

Table S9. Associations between adiposity indicators and rheumatoid arthritis incidence in UK Women's Cohort Study participants, stratified by AHEI-2010.

Adiposity Indicator	AHEI-2010 tertiles			*P*-interaction
	Low (83/9267)	Moderate (86/9356)	High (86/9345)	
**BMI (per 2.5 kg/m^2 increment)**	1.05(0.93, 1.19)	1.11(1.00, 1.22)	1.18(1.05, 1.32)	0.3
**WC (per 5 cm increment)**	1.07(0.96, 1.19)	1.10(0.99, 1.22)	1.09(0.97, 1.23)	0.7
**WHR (per 0.1 increment)**	1.17(0.91, 1.51)	1.29(0.87, 1.90)	1.22(0.93, 1.59)	0.8
**WHtR (per 0.1 increment)**	1.24(0.97, 1.68)	1.36(0.99, 1.87)	1.48(1.06, 2.05)	0.5
**Blouse size (per one-size increase)**	1.14(0.98, 1.33)	1.17(1.03, 1.34)	1.15(0.98, 1.36)	0.8
**Skirt size (per one-size increase)**	1.10(0.95, 1.27)	1.17(1.05, 1.31)	1.21(1.06, 1.39)	0.3

Models were adjusted for age, socio-economic status, marital status, menopausal status, hormone replacement therapy, and prevalence of CVD, cancer, or diabetes at recruitment, physical activity, smoking status, alcohol consumption, total energy intake; HR (95% CI), hazard ratio (95% confidence interval).

Table S10. Associations between adiposity indicators and rheumatoid arthritis incidence in UK Women's Cohort Study participants, stratified by physical activity.

Adiposity Indicator	METs (min/week)			*P*-interaction
	Low(< 600) (71/8665)	Moderate(600-1500) (148/14352)	High(> 1500) (36/4951)	
**BMI (per 2.5 kg/m² increment)**	1.08(0.97, 1.21)	1.11(1.01, 1.22)	1.19(1.03, 1.37)	0.5
**WC (per 5 cm increment)**	1.07(0.94, 1.20)	1.08(1.00, 1.19)	1.13(0.96, 1.33)	0.7
**WHR (per 0.1 increment)**	1.08(0.74, 1.58)	1.26(1.04, 1.53)	1.30(0.86, 1.96)	0.8
**WHtR (per 0.1 increment)**	1.22(0.85, 1.74)	1.37(1.08, 1.74)	1.56(1.00, 2.43)	0.6
**Blouse size (per one-size increase)**	1.14(0.98, 1.33)	1.17(1.04, 1.31)	1.13(0.88, 1.45)	0.9
**Skirt size (per one-size increase)**	1.16(1.02, 1.32)	1.15(1.04, 1.28)	1.18(0.96, 1.44)	0.8

Models were adjusted for age, socio-economic status, marital status, menopausal status, hormone replacement therapy, and prevalence of CVD, cancer, or diabetes at recruitment, smoking status, alcohol consumption, total energy intake, and Alternate Healthy Eating Index -2010; HR (95% CI), hazard ratio (95% confidence interval).

Table S11. Associations between adiposity indicators and rheumatoid arthritis incidence in UK Women's Cohort Study participants, stratified by menopausal status.

**Adiposity Indicator**	**Menopausal status**		***P*-interaction**
	**Pre-menopausal (93/13571)**	**Post-menopausal (162/14397)**	
**BMI (per 2.5 kg/m^2 increment)**	**1.22(1.13, 1.33)**	1.02(0.94, 1.11)	**0.003**
**WC (per 5 cm increment)**	**1.17(1.06, 1.29)**	1.03(0.95, 1.12)	**0.04**
**WHR (per 0.1 increment)**	1.37(0.98, 1.91)	1.16(0.94, 1.43)	0.4
**WHtR (per 0.1 increment)**	**1.70(1.35, 2.14)**	1.13(0.88, 1.45)	**0.02**
**Blouse size (per one-size increase)**	1.19(1.02, 1.38)	1.12(1.01, 1.25)	0.5
**Skirt size (per one-size increase)**	1.24(1.10, 1.40)	1.10(1.01, 1.21)	0.1

Models were adjusted for age, socio-economic status, marital status, hormone replacement therapy, and prevalence of CVD, cancer, or diabetes at recruitment, physical activity, smoking status, alcohol consumption, total energy intake, and Alternate Healthy Eating Index -2010; HR (95% CI), hazard ratio (95% confidence interval).

Table S12. Associations between adiposity indicators and rheumatoid arthritis incidence in UK Women's Cohort Study participants, stratified by smoking status.

Adiposity Indicator	Smoking status		*P*-interaction
	Smoker (130/11803)	Non-smoker (125/16165)	
**BMI (per 2.5 kg/m^2 increment)**	1.10(1.02, 1.20)	1.11(1.00, 1.23)	0.9
**WC (per 5 cm increment)**	1.07(0.98, 1.16)	1.09(0.99, 1.21)	0.7
**WHR (per 0.1 increment)**	1.17(0.95, 1.44)	1.24(0.93, 1.67)	0.7
**WHtR (per 0.1 increment)**	1.29(1.03, 1.63)	1.37(1.02, 1.83)	0.8
**Blouse size (per one-size increase)**	1.12(1.00,1.25)	1.17(1.03, 1.35)	0.6
**Skirt size (per one-size increase)**	1.15(1.05, 1.27)	1.15(1.02, 1.29)	0.9

Models were adjusted for age, socio-economic status, marital status, hormone replacement therapy, and prevalence of CVD, cancer, or diabetes at recruitment, physical activity, smoking status, alcohol consumption, total energy intake, and Alternate Healthy Eating Index -2010; HR (95% CI), hazard ratio (95% confidence interval).

Table S13. Associations between adiposity indicators and rheumatoid arthritis incidence in UK Women's Cohort Study participants, stratified by age.

Adiposity Indicator	Category	Age(years)		*P*-interaction
		< 55	≥ 55	
		(142/18252)	(113/9716)	
BMI (kg/m^2)	Normal (18.5-< 25)	1(ref)	1(ref)	0.3
	Overweight (25.0-< 30.0)	1.44(0.98, 2.12)	1.09(0.72, 1.65)	
	Obese (≥ 30.0)	2.11(1.31, 3.42)	1.19(0.68, 2.10)	
WC (cm)	Normal (≤ 88)	1(ref)	1(ref)	0.3
	Abdominal obese (> 88)	2.06(1.24, 3.41)	1.41(0.87, 2.28)	
WHR	≤ 0.8	1(ref)	1(ref)	0.1
	0.8-0.85	1.89(1.20, 2.97)	0.96(0.55, 1.66)	
	> 0.85	2.12(1.22, 3.72)	1.30(0.74, 2.28)	
WHtR	Normal (< 0.5)	1(ref)	1(ref)	0.1
	Overweight (0.5-< 0.6)	1.84(1.22, 2.78)	1.01(0.66, 1.56)	
	Obese (≥ 0.6)	3.52(1.71, 7.22)	1.76(0.85, 3.64)	
Blouse size	≤ 10	1(ref)	1(ref)	0.2
	12	1.12(0.64, 1.95)	0.76(0.37, 1.54)	
	14	1.20(0.68, 2.13)	0.49(0.24, 1.01)	
	16	1.93(1.07, 3.50)	0.69(0.34, 1.41)	
	18	1.60(0.72, 3.55)	0.95(0.44, 2.03)	
	≥ 20	2.81(1.32, 5.96)	1.00(0.45, 2.23)	
Skirt size	≤ 10	1(ref)	1(ref)	0.3

**12**	1.22(0.63, 2.36)	1.78(0.52, 6.04)
**14**	1.43(0.75, 2.74)	1.59(0.49, 5.22)
**16**	2.43(1.26, 4.67)	1.49(0.45, 4.90)
**18**	1.61(0.69, 3.72)	1.29(0.37, 4.51)
**≥ 20**	3.13(1.42, 6.88)	2.45(0.73, 8.21)

Models were adjusted for socio-economic status, marital status, hormone replacement therapy, menopausal status, and prevalence of CVD, cancer, or diabetes at recruitment, physical activity, smoking status, alcohol consumption, total energy intake, and Alternate Healthy Eating Index -2010; HR (95% CI), hazard ratio (95% confidence interval).

Table S14. Associations between adiposity indicators and rheumatoid arthritis incidence in UK Women's Cohort Study participants, stratified by weight change.

Adiposity Indicator	Category	Weight change (kg)				*P*-interaction
		Weight loss (< -2.5) (31/4147)	Stable weight (±2.5) (26/2909)	Weight gain, moderate (2.5-< 10) (98/10657)	Weight gain, large (≥ 10) (108/10255)	
**BMI (kg/m²)**	**Normal (18.5-< 25)**	1(ref)	1(ref)	1(ref)	1(ref)	0.3
	**Overweight (25.0-< 30.0)**	1.87(0.43, 8.18)	0.89(0.21, 3.80)	1.47(0.92, 2.35)	0.92(0.58, 1.46)	
	**Obese (≥ 30.0)**	N/A	3.60(0.48, 26.9)	N/A	1.43(0.88, 2.33)	
**WC (cm)**	**Normal (≤ 88)**	1(ref)	1(ref)	1(ref)	1(ref)	<0.001
	**Abdominal obese (> 88)**	N/A	N/A	0.72(0.18, 2.94)	1.87(1.24, 2.82)	
**WHR**	**≤ 0.8**	1(ref)	1(ref)	1(ref)	1(ref)	<0.001
	**0.8-0.85**	N/A	1.10(0.25, 4.83)	0.85(0.41, 1.78)	2.12(1.34, 3.35)	
	**> 0.85**	N/A	N/A	0.93(0.34, 2.55)	2.32(1.44, 3.78)	
**WHtR**	**Normal (< 0.5)**	1(ref)	1(ref)	1(ref)	1(ref)	0.4
	**Overweight (0.5-< 0.6)**	N/A	0.96(0.13, 7.38)	0.88(0.44, 1.78)	1.77(1.17, 2.69)	
	**Obese (≥ 0.6)**	N/A	N/A	N/A	3.17(1.78, 5.67)	
**Blouse size**	**≤ 10**	1(ref)	1(ref)	1(ref)	1(ref)	<0.001
	**12**	0.37(0.15, 0.90)	0.51(0.18, 1.45)	1.71(0.72, 4.10)	N/A	
	**14**	0.35(0.11, 1.17)	0.70(0.21, 2.27)	1.73(0.71, 4.21)	N/A	
	**16**	0.87(0.23, 3.36)	2.91(0.88, 9.59)	1.68(0.63, 4.47)	N/A	

	**18**	1.45(0.16, 12.80)	3.25(0.43, 24.66)	2.62(0.79, 8.67)	N/A	
	**≥ 20**	3.96(0.46, 33.50)	4.41(0.59, 33.23)	3.42(0.70, 16.84)	N/A	
**Skirt size**	**≤ 10**	1(ref)	1(ref)	1(ref)	1(ref)	<0.001
	**12**	0.86(0.32, 2.24)	1.80(0.56, 5.77)	1.39(0.48, 4.00)	N/A	
	**14**	1.16(0.40, 3.35)	1.66(0.46, 5.99)	1.53(0.54, 4.34)	N/A	
	**16**	1.12(0.26, 4.78)	3.99(0.92, 17.28)	2.10(0.72, 6.09)	N/A	
	**18**	1.57(0.17, 14.68)	3.23(0.37, 28.55)	1.18(0.30, 4.66)	N/A	
	**≥ 20**	2.77(0.30, 25.80)	4.82(0.55, 42.02)	4.23(1.15, 15.57)	N/A	

Models were adjusted for age, socio-economic status, marital status, hormone replacement therapy, menopausal status, and prevalence of CVD, cancer, or diabetes at recruitment, physical activity, smoking status, alcohol consumption, total energy intake, and Alternate Healthy Eating Index -2010; HR (95% CI), hazard ratio (95% confidence interval). N/A indicates a insufficient sample size for a reliable estimate.

Table S15. Associations between adiposity indicators and rheumatoid arthritis incidence in UK Women's Cohort Study participants, stratified by AHEI-2010.

Adiposity Indicator	Category	AHEI-2010 tertiles			*P*-interaction
		Low (83/9267)	Moderate (86/9356)	High (86/9345)	
**BMI (kg/m²)**	**Normal (18.5-< 25)**	1(ref)	1(ref)	1(ref)	0.7
	**Overweight (25.0-< 30.0)**	1.14(0.69, 1.91)	1.09(0.67, 1.79)	1.63(1.01, 2.63)	
	**Obese (≥ 30.0)**	1.46(0.78, 2.73)	1.94(1.08, 3.50)	1.45(0.68, 3.07)	
**WC (cm)**	**Normal (≤ 88)**	1(ref)	1(ref)	1(ref)	0.5
	**Abdominal obese (> 88)**	1.42(0.76, 2.64)	1.64(0.90, 2.98)	2.09(1.11, 3.91)	
**WHR**	**≤ 0.8**	1(ref)	1(ref)	1(ref)	0.4
	**0.8-0.85**	1.25(0.67, 2.34)	1.74(1.00, 3.04)	1.16(0.59, 2.27)	
	**> 0.85**	1.32(0.65, 2.67)	1.45(0.69, 3.05)	2.45(1.28, 4.71)	
**WHtR**	**Normal (< 0.5)**	1(ref)	1(ref)	1(ref)	0.9
	**Overweight (0.5-< 0.6)**	1.27(0.74, 2.19)	1.47(0.87, 2.46)	1.38(0.80, 2.39)	
	**Obese (≥ 0.6)**	2.71(1.21, 6.08)	1.78(0.64, 4.92)	3.04(1.22, 7.64)	
**Blouse size**	**≤ 10**	1(ref)	1(ref)	1(ref)	0.8
	**12**	1.21(0.57, 2.59)	1.19(0.50, 2.82)	0.75(0.38, 1.51)	
	**14**	0.78(0.35, 1.79)	1.38(0.59, 3.22)	0.68(0.32, 1.45)	
	**16**	1.09(0.47, 2.54)	1.84(0.76, 4.48)	1.21(0.57, 2.55)	
	**18**	1.03(0.38, 2.79)	2.20(0.84, 5.79)	1.41(0.57, 3.48)	
	**≥ 20**	2.13(0.86, 5.26)	2.38(0.85, 6.68)	1.29(0.44, 3.78)	
**Skirt size**	**≤ 10**	1(ref)	1(ref)	1(ref)	0.4

**12**	1.42(0.57, 3.54)	1.27(0.42, 3.88)	1.43(0.53, 3.87)
**14**	1.21(0.49, 3.00)	1.88(0.66, 5.38)	1.56(0.58, 4.20)
**16**	1.02(0.38, 2.70)	2.30(0.79, 6.75)	2.99(0.40, 4.62)
**18**	1.10(0.37, 3.26)	2.04(0.65, 6.49)	1.36(0.40, 4.62)
**≥ 20**	2.15(0.80, 5.81)	3.33(1.06, 10.42)	3.21(1.04, 9.96)

Models were adjusted for age, socio-economic status, marital status, hormone replacement therapy, menopausal status, and prevalence of CVD, cancer, or diabetes at recruitment, physical activity, smoking status, alcohol consumption, total energy intake; HR (95% CI), hazard ratio (95% confidence interval).

Table S16. Associations between adiposity indicators and rheumatoid arthritis incidence in UK Women's Cohort Study participants, stratified by physical activity.

Adiposity Indicator	Category	METs (min/week)			*P*-interaction
		Low(< 600) (71/8665)	Moderate(600-1500) (148/14352)	High(> 1500) (36/4951)	
BMI (kg/m²)	Normal (18.5-< 25)	1(ref)	1(ref)	1(ref)	0.9
	Overweight (25.0-< 30.0)	1.44(0.85, 2.44)	1.16(0.79, 1.70)	1.43(0.21,12.02)	
	Obese (≥ 30.0)	1.37(0.68, 2.79)	1.61(0.97, 2.66)	2.59(1.08, 6.26)	
WC (cm)	Normal (≤ 88)	1(ref)	1(ref)	1(ref)	0.9
	Abdominal obese (> 88)	1.49(0.77, 2.87)	1.82(1.13, 2.92)	1.56(0.61, 4.00)	
WHR	≤ 0.8	1(ref)	1(ref)	1(ref)	0.9
	0.8-0.85	1.63(0.86, 3.08)	1.26(0.78, 2.05)	1.51(0.61, 3.73)	
	> 0.85	2.01(1.01, 4.01)	1.65(0.95, 2.84)	1.14(0.34, 3.80)	
WHtR	Normal (< 0.5)	1(ref)	1(ref)	1(ref)	0.8
	Overweight (0.5-< 0.6)	1.64(0.95, 2.81)	1.35(0.89, 2.05)	0.95(0.38, 2.33)	
	Obese (≥ 0.6)	1.81(0.64, 5.13)	2.76(1.38, 5.51)	2.94(0.88, 9.84)	
Blouse size	≤ 10	1(ref)	1(ref)	1(ref)	0.9
	12	1.56(0.58, 4.23)	0.94(0.54, 1.65)	0.76(0.28, 2.10)	
	14	1.39(0.49, 3.88)	0.87(0.49, 1.56)	0.55(0.18, 1.66)	
	16	1.75(0.60, 5.15)	1.37(0.75, 2.49)	0.83(0.27,2.61)	
	18	2.61(0.84, 8.08)	1.39(0.68, 2.83)	0.54(0.12, 2.68)	
	≥ 20	2.25(0.65, 7.77)	1.81(0.86, 3.82)	1.87(0.56, 6.27)	

**Skirt size**	**≤ 10**	1(ref)	1(ref)	1(ref)	0.9
	**12**	2.34(0.52, 10.48)	1.16(0.59, 2.30)	1.61(0.33, 7.74)	
	**14**	2.62(0.59, 11.68)	1.16(0.59, 2.27)	2.45(0.56, 10.82)	
	**16**	3.88(0.86, 17.45)	1.53(0.76, 3.09)	2.37(0.52, 10.91)	
	**18**	3.59(0.73, 17.54)	1.25(0.56, 2.79)	2.34(0.51, 10.87)	
	**≥ 20**	4.33(0.86, 21.72)	2.30(1.08, 4.91)	4.44(0.91, 21.54)	

Models were adjusted for age, socio-economic status, marital status, hormone replacement therapy, menopausal status, and prevalence of CVD, cancer, or diabetes at recruitment, smoking status, alcohol consumption, total energy intake, and Alternate Healthy Eating Index -2010; HR (95% CI), hazard ratio (95% confidence interval).

Table S17. Associations between adiposity indicators and rheumatoid arthritis incidence in UK Women's Cohort Study participants, stratified by smoking status.

Adiposity Indicator	Category	Smoking status		*P*-interaction
		**Smoker (71/8665)**	**Non-smoker (148/14352)**	
**BMI (kg/m²)**	**Normal (18.5-< 25)**	1(ref)	1(ref)	0.7
	**Overweight (25.0-< 30.0)**	1.30(0.87, 1.94)	1.22(0.82, 1.84)	
	**Obese (≥ 30.0)**	1.78(1.10, 2.91)	1.37(0.77, 2.44)	
**WC (cm)**	**Normal (≤ 88)**	1(ref)	1(ref)	0.5
	**Abdominal obese (> 88)**	1.45(0.89, 2.38)	1.84(1.10, 3.09)	
**WHR**	**≤ 0.8**	1(ref)	1(ref)	0.9
	**0.8-0.85**	1.32(0.81, 2.1)	1.42(0.85, 2.38)	
	**> 0.85**	1.58(0.92, 2.71)	1.64(0.89, 3.01)	
**WHtR**	**Normal (< 0.5)**	1(ref)	1(ref)	0.3
	**Overweight (0.5-< 0.6)**	1.56(1.04, 2.34)	1.10(0.68, 1.79)	
	**Obese (≥ 0.6)**	1.85(0.85, 4.04)	3.08(1.53, 6.18)	
**Blouse size**	**≤ 10**	1(ref)	1(ref)	0.7
	**12**	0.98(0.52, 1.83)	1.02(0.55, 1.87)	
	**14**	0.71(0.36, 1.39)	1.06(0.57, 1.98)	

	**16**	1.44(0.75, 2.75)	1.10(0.55, 2.20)	
	**18**	1.32(0.62, 2.85)	1.46(0.67, 3.17)	
	**≥ 20**	1.64(0.75, 3.58)	1.97(0.87, 4.43)	
**Skirt size**	**≤ 10**	1(ref)	1(ref)	0.8
	**12**	0.93(0.43, 2.03)	2.05(0.85, 4.92)	
	**14**	1.24(0.59, 2.60)	1.92(0.81, 4.58)	
	**16**	1.69(0.80, 3.59)	2.31(0.94, 5.66)	
	**18**	1.26(0.53, 3.02)	1.70(0.62, 4.66)	
	**≥ 20**	2.24(0.99, 5.07)	3.40(1.29, 8.91)	

Models were adjusted for age, socio-economic status, marital status, hormone replacement therapy, menopausal status, and prevalence of CVD, cancer, or diabetes at recruitment, physical activity, alcohol consumption, total energy intake, and Alternate Healthy Eating Index -2010; HR (95% CI), hazard ratio (95% confidence interval).

Table S18. Associations between adiposity indicators and rheumatoid arthritis incidence in UK Women's Cohort Study participants, stratified by menopausal status.

Adiposity Indicator	Category	Menopausal status		*P*-interaction
		Pre-menopausal (93/13571)	Post-menopausal (162/14397)	
**BMI (kg/m²)**	**Normal (18.5-< 25)**	1(ref)	1(ref)	0.2
	**Overweight (25.0-< 30.0)**	1.57(0.97, 2.54)	1.10(0.78, 1.56)	
	**Obese (≥ 30.0)**	2.58(1.45, 4.58)	1.22(0.76, 1.96)	
**WC (cm)**	**Normal (≤ 88)**	1(ref)	1(ref)	0.2
	**Abdominal obese (> 88)**	1.41(0.92, 2.16)	2.40(1.31, 4.39)	
**WHR**	**≤ 0.8**	1(ref)	1(ref)	0.1
	**0.8-0.85**	1.11(0.70, 1.76)	2.02(1.18, 3.49)	
	**> 0.85**	1.35(0.82, 2.23)	2.46(1.27, 4.77)	
**WHtR**	**Normal (< 0.5)**	1(ref)	1(ref)	**0.03**
	**Overweight (0.5-< 0.6)**	1.08(0.74, 1.57)	2.12(1.28, 3.51)	
	**Obese (≥ 0.6)**	1.78(0.93, 3.41)	4.59(2.01, 10.47)	
**Blouse size**	**≤ 10**	1(ref)	1(ref)	0.5
	**12**	1.07(0.58, 1.98)	0.88(0.47, 1.65)	

	**14**	0.70(0.37, 1.33)	1.16(0.61, 2.19)	
	**16**	1.20(0.64, 2.26)	1.31(0.63, 2.73)	
	**18**	1.28(0.64, 2.26)	1.51(0.58, 3.91)	
	**≥ 20**	1.60(0.79, 3.29)	2.17(0.84, 5.63)	
**Skirt size**	**≤ 10**	1(ref)	1(ref)	0.4
	**12**	1.68(0.70, 4.03)	1.07(0.49, 2.35)	
	**14**	1.55(0.66, 3.65)	1.41(0.66, 3.00)	
	**16**	1.66(0.70, 3.95)	2.48(1.15, 5.35)	
	**18**	1.45(0.57, 3.66)	1.27(0.42, 3.80)	
	**≥ 20**	2.57(1.04, 6.30)	3.08(1.19, 7.95)	

Models were adjusted for age, socio-economic status, marital status, hormone replacement therapy, and prevalence of CVD, cancer, or diabetes at recruitment, smoking status, physical activity, alcohol consumption, total energy intake, and Alternate Healthy Eating Index -2010; HR (95% CI), hazard ratio (95% confidence interval).

Table S19. Risk of rheumatoid arthritis by adiposity indicators with varying restrictions in the UKWCS.

Adiposity indicator	Category	HR (95% CI)	*P*
**Fully-adjusted models (255 cases/ 27,968 participants)[a]**			
**BMI (per 2.5 kg/m^2 increment)**		1.09(1.02, 1.16)	< 0.01
**WC (per 5 cm increment)**		1.07(1.00, 1.13)	0.04
**WHR (per 0.1 increment)**		1.20(1.01, 1.43)	0.04
**WHtR (per 0.1 increment)**		1.27(1.05, 1.53)	0.01
**Blouse size (per one-size increase)**		1.13(1.04, 1.22)	< 0.01
**Skirt size (per one-size increase)**		1.13(1.05, 1.22)	< 0.01
**BMI (kg/m^2)**	**Normal (18.5-< 25)**	1(ref)	
	**Overweight (25.0-< 30.0)**	1.27(0.95, 1.70)	0.11
	**Obese (≥ 30.0)**	1.49(1.02, 2.18)	0.04
**WC (cm)**	**Normal (≤ 88)**	1(ref)	
	**Abdominal obese (> 88)**	1.59(1.10, 2.28)	0.01
**WHR**	**≤ 0.8**	1(ref)	
	**0.8-0.85**	1.38(0.97, 1.97)	0.08
	**> 0.85**	1.56(1.03, 2.36)	0.04
**WHtR**	**Normal (< 0.5)**	1(ref)	
	**Overweight (0.5-< 0.6)**	1.26(0.92, 1.73)	0.15
	**Obese (≥ 0.6)**	2.26(1.34, 3.81)	< 0.01
**Blouse size**	**≤ 10**	1(ref)	
	**12**	0.97(0.62, 1.52)	0.89

	**14**	0.87(0.55, 1.37)	0.54
	**16**	1.24(0.77, 1.99)	0.37
	**18**	1.28(0.73, 2.23)	0.39
	**≥ 20**	1.64(0.93, 2.89)	0.09
**Skirt size**	**≤ 10**	1(ref)	
	**12**	1.35(0.76, 2.40)	0.31
	**14**	1.42(0.81, 2.50)	0.23
	**16**	1.73(0.97, 3.08)	0.06
	**18**	1.33(0.68, 2.58)	0.41
	**≥ 20**	2.36(1.25, 4.45)	< 0.01
**Excluding participants with survival time < 5 years (255 cases/ 27,968 participants)**			
**BMI (per 2.5 kg/m² increment)**		1.08(1.01, 1.16)	**0.02**
**WC (per 5 cm increment)**		1.08(1.00, 1.16)	**0.04**
**WHR (per 0.1 increment)**		1.21(1.00, 1.48)	**0.05**
**WHtR (per 0.1 increment)**		1.30(1.07, 1.59)	**< 0.01**
**Blouse size (per one-size increase)**		1.14(1.04, 1.26)	**< 0.01**
**Skirt size (per one-size increase)**		1.14(1.05, 1.24)	**< 0.01**
**BMI (kg/m²)**	**Normal (18.5-< 25)**	1(ref)	
	**Overweight (25.0-< 30.0)**	1.30(0.95, 1.78)	0.09
	**Obese (≥ 30.0)**	1.49(0.98,2.25)	0.06
**WC (cm)**	**Normal (≤ 88)**	1(ref)	
	**Abdominal obese (> 88)**	1.55(1.03, 2.32)	0.04

**WHR**	**≤ 0.8**	1(ref)	
	**0.8-0.85**	1.38(0.93, 2.03)	0.11
	**> 0.85**	1.51(0.94, 2.40)	0.09
**WHtR**	**Normal (< 0.5)**	1(ref)	
	**Overweight (0.5-< 0.6)**	1.31(0.92, 1.86)	0.14
	**Obese (≥ 0.6)**	2.29(1.27, 4.13)	**< 0.01**
**Blouse size**	**≤ 10**	1(ref)	
	**12**	1.13(0.69, 1.86)	0.63
	**14**	0.94(0.56, 1.58)	0.82
	**16**	1.41(0.83, 2.40)	0.20
	**18**	1.54(0.83, 2.86)	0.18
	**≥ 20**	1.90(1.01, 3.56)	**0.05**
**Skirt size**	**≤ 10**	1(ref)	
	**12**	1.43(0.75, 2.73)	0.28
	**14**	1.71(0.91, 3.20)	0.09
	**16**	1.75(0.91, 3.38)	0.09
	**18**	1.73(0.84, 3.57)	0.14
	**≥ 20**	2.68(1.33, 5.43)	**< 0.01**
**Excluding participants who are current smoker (255 cases/ 27, 968 participants)**			
**BMI (per 2.5 kg/m^2 increment)**		**1.13(1.06, 1.21)**	**< 0.01**
**WC (per 5 cm increment)**		1.11(1.04, 1.19)	**< 0.01**
**WHR (per 0.1 increment)**		1.30(1.11, 1.53)	**< 0.01**

**WHtR (per 0.1 increment)**		1.42(1.17, 1.74)	**< 0.01**
**Blouse size (per one-size increase)**		1.17(1.07, 1.29)	**< 0.01**
**Skirt size (per one-size increase)**		1.17(1.08, 1.28)	**< 0.001**
**BMI (kg/m²)**	**Normal (18.5-< 25)**	1(ref)	
	**Overweight (25.0-< 30.0)**	1.45(1.05, 1.98)	**0.02**
	**Obese (≥ 30.0)**	1.77(1.19,2.66)	**< 0.01**
**WC (cm)**	**Normal (≤ 88)**	1(ref)	
	**Abdominal obese (> 88)**	1.84(1.26, 2.70)	< 0.01
**WHR**	**≤ 0.8**	1(ref)	
	**0.8-0.85**	1.48(1.00, 2.18)	**0.05**
	**> 0.85**	1.80(1.15, 2.80)	**< 0.01**
**WHtR**	**Normal (< 0.5)**	1(ref)	
	**Overweight (0.5-< 0.6)**	1.44(1.01, 2.03)	**0.04**
	**Obese (≥ 0.6)**	2.50(1.42, 4.40)	**< 0.01**
**Blouse size**	**≤ 10**	1(ref)	
	**12**	0.90(0.55, 1.48)	0.67
	**14**	0.85(0.51, 1.42)	0.54
	**16**	1.43(0.85, 2.39)	0.17
	**18**	1.32(0.71, 2.42)	0.38
	**≥ 20**	1.96(1.07, 3.59)	**0.03**
**Skirt size**	**≤ 10**	1(ref)	
	**12**	1.07(0.57, 2.01)	0.84

	**14**	1.30(0.71, 2.39)	0.71
	**16**	1.76(0.94, 3.28)	0.08
	**18**	1.21(0.59, 2.49)	0.60
	**≥ 20**	2.60(1.33, 5.10)	**< 0.01**
**Excluding participants who developed chronic diseases at recruitment (255 cases/ 27, 968 participants)**			
BMI (per 2.5 kg/m^2 increment)		1.09(1.01,1.17)	**0.02**
WC (per 5 cm increment)		1.07(1.00, 1.15)	**0.05**
WHR (per 0.1 increment)		1.20(0.99, 1.45)	0.07
WHtR (per 0.1 increment)		1.31(1.07, 1.62)	**0.01**
Blouse size (per one-size increase)		1.15(1.05, 1.27)	**< 0.01**
Skirt size (per one-size increase)		1.15(1.05, 1.25)	**< 0.01**
**BMI (kg/m^2)**	**Normal (18.5-< 25)**	1(ref)	
	**Overweight (25.0-< 30.0)**	1.28(0.94, 1.75)	0.11
	**Obese (≥ 30.0)**	1.57(1.04, 2.36)	0.03
**WC (cm)**	**Normal (≤ 88)**	1(ref)	
	**Abdominal obese (> 88)**	1.60(1.07, 2.39)	**0.02**
**WHR**	**≤ 0.8**	1(ref)	
	**0.8-0.85**	1.30(0.88, 1.94)	0.19
	**> 0.85**	1.63(1.04, 2.55)	**0.03**
**WHtR**	**Normal (< 0.5)**	1(ref)	
	**Overweight (0.5-< 0.6)**	1.19(0.83, 1.69)	0.35
	**Obese (≥ 0.6)**	2.69(1.56, 4.65)	**< 0.001**

**Blouse size**	**≤ 10**	1(ref)	
	**12**	0.91(0.56, 1.48)	0.71
	**14**	0.97(0.59, 1.58)	0.90
	**16**	1.20(0.72, 2.01)	0.49
	**18**	1.40(0.77, 2.55)	0.27
	**≥ 20**	1.67(0.90, 3.12)	0.11
**Skirt size**	**≤ 10**	1(ref)	
	**12**	1.34(0.72, 2.50)	0.36
	**14**	1.50(0.81, 2.76)	0.19
	**16**	1.75(0.93, 3.30)	0.08
	**18**	1.54(0.75, 3.13)	0.24
	**≥ 20**	2.50(1.25, 4.99)	**< 0.01**
**Excluding participants with weight change of more than 10 kg (255 cases/ 27, 968 participants)**			
BMI (per 2.5 kg/m^2 increment)		0.94(0.83, 1.06)	0.30
WC (per 5 cm increment)		1.03(0.91, 1.17)	0.60
WHR (per 0.1 increment)		0.81(0.59,1.12)	0.20
WHtR (per 0.1 increment)		0.88(0.61, 1.27)	0.50
Blouse size (per one-size increase)		1.23(1.03, 1.46)	0.02
Skirt size (per one-size increase)		1.19(1.04, 1.36)	0.01
**BMI (kg/m^2)**	**Normal (18.5-< 25)**	1(ref)	
	**Overweight (25.0-< 30.0)**	1.58(1.05, 2.38)	0.03
	**Obese (≥ 30.0)**	0.55(0.08, 3.95)	0.55

**WC (cm)**	**Normal (≤ 88)**	1(ref)	
	**Abdominal obese (> 88)**	0.56(0.14,2.25)	0.41
**WHR**	**≤ 0.8**	1(ref)	
	**0.8-0.85**	0.83(0.44, 1.55)	0.56
	**> 0.85**	0.72(0.27, 1.95)	0.27
**WHtR**	**Normal (< 0.5)**	1(ref)	
	**Overweight (0.5-< 0.6)**	0.82(0.43, 1.59)	0.56
	**Obese (≥ 0.6)**	N/A	N/A
**Blouse size**	**≤ 10**	1(ref)	
	**12**	0.79(0.49, 1.28)	0.33
	**14**	0.87(0.52, 1.45)	0.59
	**16**	1.25(0.69, 2.26)	0.47
	**18**	1.55(0.63, 3.79)	0.34
	**≥ 20**	2.97(1.08, 8.12)	**0.03**
**Skirt size**	**≤ 10**	1(ref)	
	**12**	1.19(0.65, 2.16)	0.57
	**14**	1.29(0.71, 2.36)	0.41
	**16**	1.70(0.88, 3.30)	0.12
	**18**	1.30(0.51, 3.35)	0.59
	**≥ 20**	3.18(1.22, 8.31)	**0.02**
**Further adjusted for dietary vitamin D (255 cases/ 27, 968 participants)**			
BMI (per 2.5 kg/m^2 increment)		1.09(1.02, 1.17)	**0.01**

WC (per 5 cm increment)		1.07(1.00, 1.14)	**0.05**
WHR (per 0.1 increment)		1.20(1.00, 1.44)	**0.05**
WHtR (per 0.1 increment)		1.27(1.05, 1.54)	**0.02**
Blouse size (per one-size increase)		1.13(1.03, 1.24)	**< 0.01**
Skirt size (per one-size increase)		1.13(1.04, 1.22)	**< 0.01**
**BMI (kg/m²)**	**Normal (18.5-< 25)**	1(ref)	
	**Overweight (25.0-< 30.0)**	1.27(0.95, 1.69)	0.11
	**Obese (≥ 30.0)**	1.48(1.02, 2.17)	**0.04**
**WC (cm)**	**Normal (≤ 88)**	1(ref)	
	**Abdominal obese (> 88)**	1.58(1.10, 2.28)	**0.01**
**WHR**	**≤ 0.8**	1(ref)	
	**0.8-0.85**	1.38(0.96, 1.97)	0.08
	**> 0.85**	1.56(1.02, 2.37)	**0.04**
**WHtR**	**Normal (< 0.5)**	1(ref)	
	**Overweight (0.5-< 0.6)**	1.26(0.91, 1.74)	0.16
	**Obese (≥ 0.6)**	1.25(1.32, 3.84)	**< 0.01**
**Blouse size**	**≤ 10**	1(ref)	
	**12**	0.97(0.62, 1.52)	0.89
	**14**	0.87(0.54, 1.38)	0.55
	**16**	1.24(0.77, 2.01)	0.38
	**18**	1.27(0.72, 2.25)	0.41
	**≥ 20**	1.64(0.93, 2.90)	0.09

**Skirt size**	**≤ 10**	1(ref)	
	**12**	1.35(0.75, 2.40)	0.32
	**14**	1.42(0.80, 2.51)	0.23
	**16**	1.73(0.96, 3.10)	0.07
	**18**	1.32(0.67, 2.60)	0.41
	**≥ 20**	2.36(1.25, 4.46)	**< 0.01**
**Used competing risk regression model (Fine-Gray method, 255 cases/ 27, 968 participants)**			
BMI (per 2.5 kg/m^2 increment)		1.09(1.02, 1.17)	0.01
WC (per 5 cm increment)		1.06(1.00, 1.14)	0.05
WHR (per 0.1 increment)		1.20(1.00, 1.44)	0.05
WHtR (per 0.1 increment)		1.27(1.05, 1.55)	0.02
Blouse size (per one-size increase)		1.13(1.03, 1.24)	< 0.01
Skirt size (per one-size increase)		1.13(1.04, 1.22)	< 0.01
**BMI (kg/m^2)**	**Normal (18.5-< 25)**	1(ref)	
	**Overweight (25.0-< 30.0)**	1.27(0.95, 1.69)	0.11
	**Obese (≥ 30.0)**	1.49(1.02, 2.17)	0.04
**WC (cm)**	**Normal (≤ 88)**	1(ref)	
	**Abdominal obese (> 88)**	1.58(1.10, 2.28)	0.01
**WHR**	**≤ 0.8**	1(ref)	
	**0.8-0.85**	1.38(0.96, 1.97)	0.08
	**> 0.85**	1.56(1.02, 2.37)	0.04
**WHtR**	**Normal (< 0.5)**	1(ref)	

	**Overweight (0.5-< 0.6)**	1.26(0.91, 1.74)	0.16
	**Obese (≥ 0.6)**	2.25(1.32, 3.84)	< 0.01
**Blouse size**	**≤ 10**	1(ref)	
	**12**	0.97(0.62, 1.52)	0.89
	**14**	0.87(0.54, 1.38)	0.55
	**16**	1.24(0.77, 2.01)	0.38
	**18**	1.27(0.72, 2.25)	0.40
	**≥ 20**	1.64(0.92. 2.91)	0.09
**Skirt size**	**≤ 10**	1(ref)	
	**12**	1.35(0.75, 2.41)	0.32
	**14**	1.42(0.80, 2.51)	0.23
	**16**	1.73(0.96, 3.11)	0.07
	**18**	1.32(0.67, 2.60)	0.42
	**≥ 20**	2.36(1.24, 4.47)	< 0.01

[a] Models were adjusted for age, socio-economic status, marital status, physical activity, smoking status, alcohol consumption, total energy intake, the Alternate Healthy Eating Index -2010, menopausal status, hormone replacement therapy, and the prevalence of CVD, cancer, or diabetes. All sensitivity analyses were based on the adjusted model. HR (95% CI): hazard ratio (95% confidence interval).

Supplementary methods:

Cox proportional hazards regression models stratified by age group

In this study, age was used as the time scale. Due to a violation of the proportional hazards assumption (assessed using Schoenfeld residuals), we stratified the Cox model by age group (≤ 55 and > 55 years). This approach adjusts for non-proportionality related to age, allowing for a single summary HR for each adiposity indicator while accounting for age-related differences in baseline risk. The updated proportional hazards test results are presented in Table A4.
